# *In silico* Identification of miRNAs and Their Targets in Cluster Bean for Their Role in Development and Physiological Responses

**DOI:** 10.3389/fgene.2022.930113

**Published:** 2022-06-30

**Authors:** Vrantika Chaudhary, Sumit Jangra, Neelam R. Yadav

**Affiliations:** Department of Molecular Biology, Biotechnology and Bioinformatics, CCS Haryana Agricultural University, Hisar, India

**Keywords:** micro RNAs (miRNAs), cluster bean (*Cyamopsis tetragonoloba* L. Taub.), miRNA identification, miRNA targets, non coding RNAs, galactomannan

## Abstract

Cluster bean popularly known as “guar” is a drought-tolerant, annual legume that has recently emerged as an economically important crop, owing to its high protein and gum content. The guar gum has wide range of applications in food, pharma, and mining industries. India is the leading exporter of various cluster bean-based products all across the globe. Non-coding RNAs (miRNAs) are involved in regulating the expression of the target genes leading to variations in the associated pathways or final protein concentrations. The understanding of miRNAs and their associated targets in cluster bean is yet to be used to its full potential. In the present study, cluster bean EST (Expressed Sequence Tags) database was exploited to identify the miRNA and their predicted targets associated with metabolic and biological processes especially response to diverse biotic and abiotic stimuli using *in silico* approach. Computational analysis based on cluster bean ESTs led to the identification of 57 miRNAs along with their targets. To the best of our knowledge, this is the first report on identification of miRNAs and their targets using ESTs in cluster bean. The miRNA related to gum metabolism was also identified. Most abundant miRNA families predicted in our study were miR156, miR172, and miR2606. The length of most of the mature miRNAs was found to be 21nt long and the range of minimal folding energy (MFE) was 5.8–177.3 (−kcal/mol) with an average value of 25.4 (−kcal/mol). The identification of cluster bean miRNAs and their targets is predicted to hasten the miRNA discovery, resulting in better knowledge of the role of miRNAs in cluster bean development, physiology, and stress responses.

## 1 Introduction

Cluster bean (*Cyamopsis tetragonoloba* L. Taub.) popularly known as guar is a drought-tolerant, large-seeded legume, mainly adapted to semi-arid parts of the world with minimal requirements. It is commonly used as a source of food and feed in the Indian subcontinent and is a good source of nutrition, as it is a rich source of dietary fiber, folic acid, and Vitamin C (Rajaprakasam et al., 2021). India accounts for 80% of the global cluster bean production making it second-largest cash crop in India (Sahu et al., 2020). In the past two decades, the demand for cluster bean production has increased and cultivation has also been initiated in non-traditional areas like the United States, Australia, Brazil, Italy, Spain, Morocco and Germany, most recently, Russia due to the discovery of a valuable polysaccharide-galactomannan (guar gum), in the seed endosperm (Teplyakova et al., 2019; Grigoreva et al., 2021a). The gel-like structure formed by hydrated gum has a wide utility in various industries like paper, food and agriculture, cosmetics, explosives, petrochemicals, and water purification where it is used as thickener, stiffener, stabilizer, and strengthening agent (Mudgil et al., 2014; Sharma et al., 2018; Saya et al., 2021). The demand for guar gum has increased by four times in crude oil industry since 2006 (Bhatt et al., 2016). The medicinal properties of cluster bean viz. antihyperlipidemic, antihyperglycemic, and antihypercholesterolemic have made it an important constituent of pharma industry. Recently, it has been used in nanotechnology-based drug delivery and as binder in electrochemical industry (George et al., 2019; Verma and Sharma, 2021; Kaur and Santra, 2022). The global demands for cluster bean in various industries have increased several folds in the past decade making it a crop of high economic importance.

Despite such high economic importance this crop has not been explored and characterized to its full extent at genomic level. Though, recently some studies have been reported (Kaila et al., 2017; Rawal et al., 2017; Tanwar et al., 2017; Al-Qurainy et al., 2019; Chaudhury et al., 2019; Sahu et al., 2020; Rajaprakasam et al., 2021) and the availability of the whole genome sequence which identified 34,680 protein-coding genes from 550.31 Mbp of genome will further help in accelerating the fundamental and applied research in this crop (Gaikwad et al., 2020 Unpublished). When it comes to the non-coding genome, especially microRNA the research is still at its initial stages and yet to be explored to its full potential. miRNAs are single-stranded RNAs with a length of 21–25 nucleotides (nt) that are formed from stem-loop precursors and are highly conserved (Carthew and Sontheimer, 2009; O'Brien et al., 2018). These small non-coding RNAs (miRNAs) are involved in regulation of growth, development, and metabolic pathways. In Leguminosae family, a wide range of miRNAs has been reported from nine species (Kozomara and Griffiths-Jones, 2014). miRNAs and their precursors in response to various physiological stresses have also been reported in important cereals like rice, wheat, maize, sorghum, and barley (Budak and Akpinar, 2015; Djami-Tchatchou et al., 2017; Singroha et al., 2021). All these studies showed that miRNAs play an important role in regulation of physiological responses, developmental and metabolic pathways, and evolution in plants.

When genome sequence is unavailable, EST (expressed sequence tags) and GSS (genome survey sequences) data can be utilized for identification of miRNA and their targets. *In silico* miRNA prediction and characterization are developing as faster and more efficient methods than laboratory-based cloning methods, as they are cheap and quick (Gupta et al., 2017). Such *in silico* methods based on EST and GSS have been used to identify miRNAs and their target in various crop plants including cotton, soybean, lentil, pepper, potato, sweet potato, tomato, tobacco, and horse gram (Qiu et al., 2007; Zhang et al., 2008; Kim et al., 2011; Budheswar et al., 2013; Dehury et al., 2013; Vivek, 2018; Yasin et al., 2020). However, no miRNAs for the cluster bean have been reported in miRBase, and these have yet to be properly studied. Though, few recent studies report the identification of various miRNA families in cluster bean (Tyagi et al., 2018; Sahu et al., 2018). In the present study, we used EST-based identification and characterization of conserved miRNAs belonging to different families and their probable target genes. Predicted miRNAs were also functionally annotated using network analysis and gene ontology. Understanding the role of miRNAs in the control of various biological processes necessitates the study of interactions between individual miRNAs and their target genes (Bansal et al., 2017). Further, the discovery of cluster bean miRNAs and their targets is predicted to hasten miRNA discovery, resulting in a better knowledge of the role of miRNAs in cluster bean development, physiology, and stress responses.

## 2 Materials and Methods

### 2.1 Sequence Collection and Software Information

A total of 16,503 EST sequences of cluster bean were downloaded from the GenBank nucleotide database (http://www.ncbi.nlm.nih.gov/nucest) available at NCBI. To identify conserved miRNAs genes and their targets, C-mii (version 1.11) programme was used (Numnark et al., 2012). To determine putative miRNA sequences, 4,256 non-redundant mature plant miRNA sequences of viridiplantae (green plants) were also downloaded from miRBASE. BLASTN with an e-value cut-off equal to 10 was performed against the EST sequence of cluster bean and mature miRNA sequences from miRBASE. Next, to remove protein-coding sequences BLASTX with e value b = 1e^−5^ was done against UniprotKB/Swissprot (release 2010-12) and UniProtKB/TrEMBL (release 2011-01) protein databases. The primary and precursor miRNAs structures were predicted using RNA database Rfam 10. UNAFold was run at a maximum base pair distance of 3,000, maximum bulge/ interior loop size 30, and single read run of 37°C temperature.

### 2.2 Prediction of the Secondary Structure of Cluster Bean miRNAs

miRNA sequences that matched at least 18 nt and had a 3 nt mismatch with all known plant mature miRNAs were chosen for further study. The top BLAST hits were collected, and the BLASTX tool was used to execute the anticipated precursor sequences. Non-coding sequences were preserved, while protein-coding precursor sequences were removed. Rfam (10) was used to differentiate miRNA from other RNA families (rRNA, snRNA, and tRNA). UNAFold was used to anticipate secondary structure using parameters such as maximum base pair distance (3,000), maximum bulge/interior loop size (30), and single tread run temperature (37°C).

Using a homology search, the following criteria were utilized to find miRNAs: 1) The predicted mature miRNAs should be 19–25 nucleotides in length, 2) Maximum of four mismatches against the reference miRNA were allowed, 3) The mature miRNA should be localized within a stem-loop structure with one arm, 4) No more than five mismatches were allowed between miRNA sequences and the guide miRNA sequence, 5) The A + U content should be high, and the secondary structure’s minimal folding free energy (MFE) and MFE index (MFEI) values should be significantly negative.

The MFEI was calculated using the following equation- MFEI=[(MFE/length of the RNA sequence)∗100]/(G+C)%

### 2.3 Prediction of miRNA Targets

**FIGURE 1:**
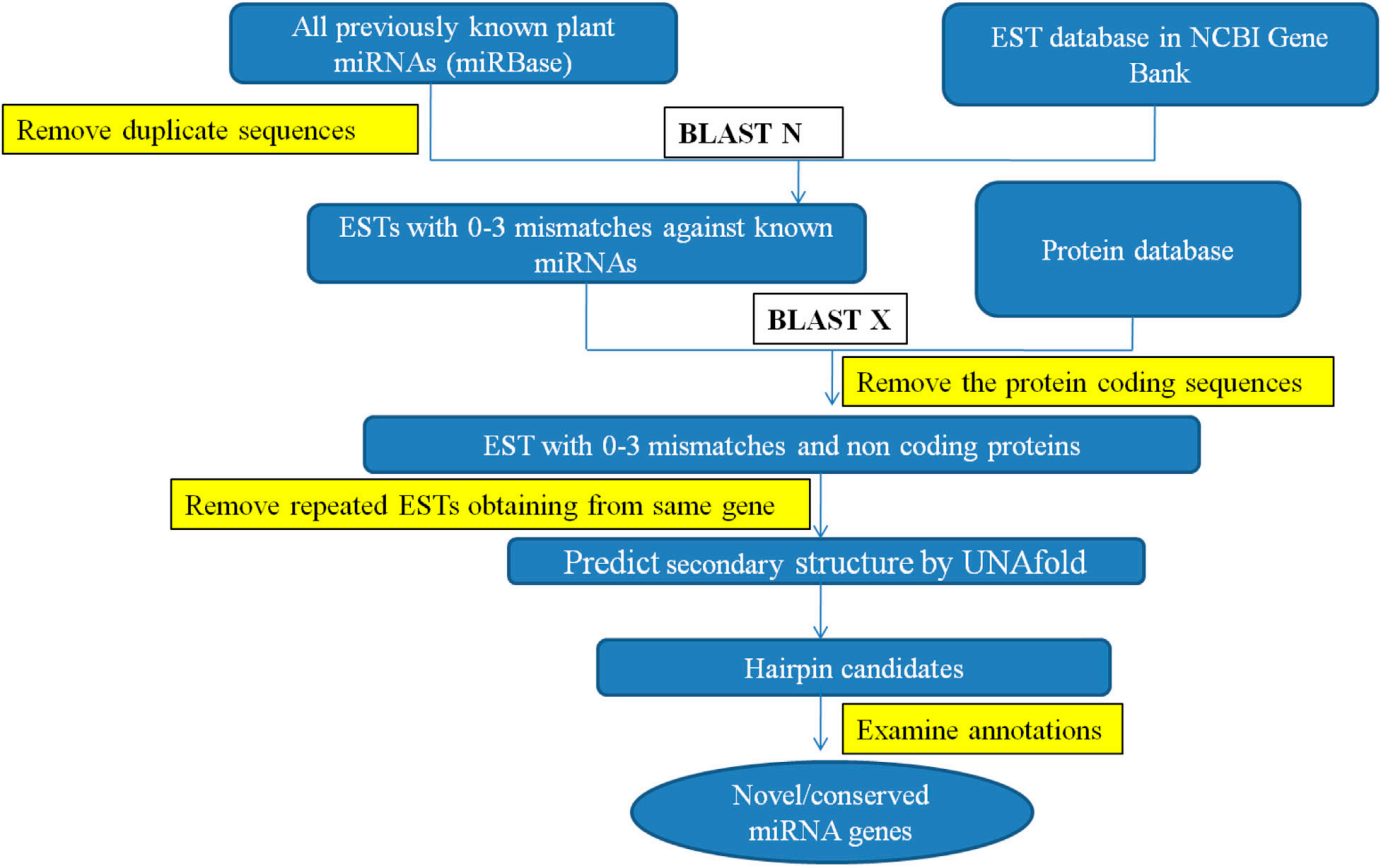
Workflow for identification of miRNA and their targets in cluster bean.

C-mii and the Plant Target Prediction Tool, both available on the UEA sRNA toolkit (srna-tools.cmp.uea.ac.uk/plant/cgi-bin/srna-tools.cgi), were used to estimate the likely target locations of detected miRNAs. miRNA-targeted mRNAs have perfectly or almost perfectly complementary sites with miRNAs and miRNAs suppress gene expression by binding to these targeted mRNAs at their complementary sites for direct mRNA cleavage or protein translation suppression (Bartel, 2004; Chen et al., 2004). This suggests that using homology search to predict miRNA targets in plants is an effective method. Target identification was carried out by comparing identified miRNA candidates to the same transcript used for miRNA identification. To predict miRNA-target genes, the following parameters were used: 1) No more than four mismatches were allowed between projected mRNAs and target genes, 2) No mismatches were allowed for the 10^th^ and 11^th^ positions of the complementary site since they are regarded as cleavage sites, and 3) Maximum of four GU pairs were allowed in the complementary alignment. All the steps involved in identification of miRNA and their targets in cluster bean are represented in Figure 1.

### 2.4 Phylogenetic Analysis of Identified miRNAs

The precursor sequences of the identified and well-known cluster bean miRNAs were aligned and phylogenetically analyzed to examine their evolutionary relationships (www.clustal.org/). The evolutionary distances were calculated using the neighbor-joining (NJ) method after 1,000 bootstrapped iterations.

### 2.5 Analysis of Gene Ontology for Predicted Targets

GO analysis of the identified target transcripts and pathway analysis was performed using the target annotation module in C-mii, which calculates and visualizes the distribution of selected targets, to better understand the role of cluster bean miRNAs and their regulating targets.

### 2.6 The miRNA-Target Gene Regulatory Network

To study co-regulated targets by miRNA families and to select miRNA-targets based on MFE value, a biological network was created between identified miRNAs and their targets. GENEMANIA (http://genemania.org/) was used to visualize the biological network of miRNA and its target.

### 2.7 Identification of Transcription Factors From Expressed Sequence Tags of Cluster Bean

Homology based search against the plant transcription factor database (PlnTFDB) was performed to identify genes encoding transcription factors in the EST of cluster bean (Pérez-Rodríguez, 2010).

## 3 Results

### 3.1 Identification of miRNA Using Expressed Sequence Tags Sequences

**FIGURE 2:**
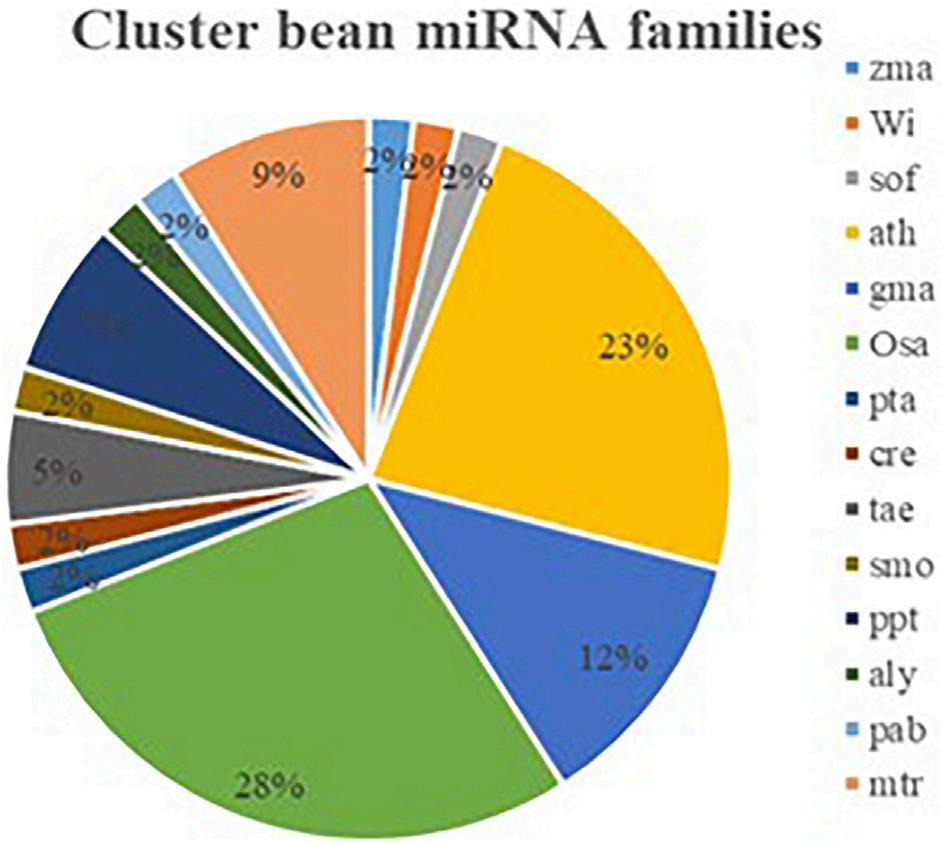
Identified miRNA families of cluster bean which have homologues in other plant species (ath- *Arabidopsis thaliana*, osa-*Oryza sativa*, gma-*Glycine max*, mtr-*Medicago truncatula*, zma-*Zea mays*, vvi-*Vitis vinifera*, sof-*Saccharum officinarum*, pab-*Picea abies*, aly-*Arabidopsis lyrata*, smo-*Selaginella moellendorffii*, tae-*Triticum aesativum*, cre-*Chlamydomonas reinhardtii*, pta- *Pinus taeda*, ppt-*Physcomitrella patens*).

To identify and characterize the conserved miRNA in cluster bean, a thorough EST-based approach using C-mii (version1.11, Numnark et al., 2012) software was employed as genomic sequence information is limited. The software includes four steps such as sequence loading, homolog search, primary miRNA folding, and precursor miRNA folding. A total of 16,503 EST sequences of cluster bean were downloaded from the public database available at (http://www.ncbi.nlm.nih.gov/nucest) and compared to the non-redundant viridiplantae mature plant miRNA sequences (4,256) retrieved from miRBase (Supplementary Figure S1). Sequences longer than 3000 nt or containing characters other than A, T, C, G, U, and N were excluded. The homolog search module scans different plant mature miRNAs from miRBase with 16,503 EST sequences with default parameter setting. Using the plus strand, only 361 miRNA families and 670 members were identified. These sequences were selected as input for the primary miRNA folding module. Next, the primary miRNA folding was done with default parameter settings except for the BLAST e-value ≤ 1E-5 which resulted in successfully folding 3,068 sequences. These sequences were again selected for the next step i.e., the precursor miRNA folding. A different number of sequences remained at each step (Supplementary Table S1). The precursor miRNA folding at default parameter settings except for the BLASTX e-value ≤ 1E-5 resulted in 74 miRNA candidates, belonging to 57 miRNA families predicted from cluster bean EST sequences available at NCBI. For reducing the duplicity and improving the accuracy of predicted miRNAs, the false-positive results only plus strand and high negative values of MFEI (b = −0.6) were considered, respectively. The predicted 57 miRNA families showing b = −0.6 MFEI value are listed in Supplementary Table S1. All potential miRNAs of cluster bean were located at the 5′ end of the precursor miRNA. Several plant species such as *Arabidopsis thaliana*, *Oryza sativa*, *Glycine max*, *Medicago truncatula*, *Zea mays*, *Vitis vinifera*, *Saccharum officinarum*, *Picea abies*, *Arabidopsis lyrata*, *Selaginella moellendorffii*, *Triticum aestivum*, *Chlamydomonas reinhardtii, Pinus taeda* were found to have miRNA family homologs in them. Out of these, *Oryza sativa* (28%), *Arabidopsis thaliana* (23%), and *Glycine max* (12%) had major miRNA family homologs followed by *Medicago truncatula* (9%; Figure 2).

miRNA families which were predicted from EST database were matched against cDNA library of seed development stage and out of 57 EST IDs in which different miRNA families were present, only 19 EST IDs were found to be involved in various functions (Supplementary Table S2).

### 3.2 Characteristics of Newly Identified Cluster Bean miRNA

To validate newly identified miRNAs, various calculated parameters were analyzed for miRNA family member, precursor length of miRNA, A + U content, G + C content, their ratios, the minimal folding free energy (MFE), and minimal folding free energy index (MFEI) for each miRNA precursor.

#### 3.2.1 miRNA Family Members

Most of the miRNA families had one member but some of the miRNAs families were observed to have more than one number. These families were miR156 (9), miR 2606 (7), and miR172 (Supplementary Figure S2).

#### 3.2.2 Precursor miRNA Length

The length of precursor miRNAs varied from 35 to 539 nt in length with an average value of 100 nt. miR1132 was found to have the lowest miRNA pre-miRNA length (35 nt) and miR1439 had highest miRNA length (539 nt; Supplementary Figure S3).

#### 3.2.3 Mature miRNA Length

The length of most of the mature miRNAs was found to be 21 nt. Based on miRNA length, the predicted miRNA was divided into three groups. These groups had 19, 20, or 21 nucleotide length in mature miRNA except in two groups. First group was 19 nt long, while second group was 20 nt long, and third group was 21 nt long (Supplementary Table S3).

#### 3.2.4 Minimal Folding Free Energy

The minimal folding free energy (MFE) has been considered one of the significant features for identification of miRNA. It is an important parameter for determining the secondary structure of pre-miRNA. Highly negative MFE indicates thermodynamically stable secondary structure of the corresponding sequences. In the present study, the range of MFE (ΔG kcal/mol) was 5.8–177.3 (−kcal/mol) with an average value of 25.4 (−kcal/mol) (Supplementary Figure S4).

#### 3.2.5 Minimal Folding Free Energy Index

The index that differentiates pre-miRNA from other coding and non-coding RNAs and RNA fragments is MFEI. The MFEI values observed in the present study ranged from 0.59 to 1.36 −kcal/mol. The mean MFEI values were found to be 0.70 −kcal/mol (Supplementary Figure S5). The MFEI values of tRNAs, rRNAs, and mRNA are 0.64, 0.59, and 0.62–0.66 −kcal/mol, respectively which are significantly lower than the cluster bean miRNAs reported in this study, signifying that the identified cluster bean miRNAs are true miRNAs.

#### 3.2.6 AU Content

The analysis also showed that AU contents in all identified miRNA precursors ranged from 41.09 to 83.12% with an average of 61.18% (Supplementary Figure S6).

#### 3.2.7 GC Content

GC content was also analyzed for different miRNA families predicted in cluster beans. The GC content ranged from 18.36 to 60.45% with an average of 38.81% (Supplementary Figure S7).

#### 3.2.8 Nucleotide Content

The nucleotide content was not uniform throughout the predicted miRNA families (Supplementary Figure S8). The A/U and C/G ratio was also calculated as given in Supplementary Table S4. The highest A/U was observed for miR1527 (1.6) and the lowest was for miRNA 444 (0.3) whereas the highest C/G ratio was for miR1527 (1.4) and the lowest for miR537 (0.2). All parameters of different characteristics of predicted miRNA families are summarized in Table 1.

**TABLE 1:** Summarized parameters of predicted miRNA families in cluster bean.

Predicted miRNA family	AU	GC	Nucleotide content	Pre-miRNA sequence length	MFE	MFEI
miR1044	70.13	29.87	A(39)U(69)G(26)C(20)N(0)	154	−30.3	−0.65
miR1109	69.93	30.07	A(39)U(54)G(23)C(17)N(0)	133	−27.8	−0.69
miR1132	62.86	37.14	A(10)U(12)G(8)C(5)N(0)	35	−12.8	−0.98
miR1134	66.08	33.92	A(20)U(17)G(9)C(10)N(0)	56	−12.5	−0.65
miR1167	58.7	41.3	A(14)U(13)G(11)C(8)N(0)	46	−16.2	−0.85
miR1313	64.9	35.1	A(26)U(35)G(17)C(16)N(0)	94	−22.9	−0.69
miR1439	80.07	19.93	A(240)U(233)G(50)C(68)N(1)	592	−75.3	−0.63
miR1527	63.05	36.95	A(18)U(11)G(7)C(10)N(0)	46	−11	−0.64
miR1533	83.12	16.88	A(35)U(29)G(8)C(5)N(0)	77	−9.9	−0.761
miR1535	42.07	57.93	A(22)U(39)G(41)C(43)N(0)	145	−51.6	−0.61
miR156	56.96	43.04	A(48)U(38)G(31)C(34)N(0)	151	−38.4	−0.59
miR168	47.92	52.08	A(12)U(11)G(13)C(12)N(0)	48	−16.6	−0.66
miR169	56.9	43.1	A(17)U(16)G(13)C(12)N(0)	58	−15.9	−0.63
miR172	62.5	37.5	A(19)U(16)G(9)C(12)N(0)	56	−14.7	−0.7
miR1852	56.1	43.9	A(19)U(26)G(22)C(14)N(1)	82	−21.9	−0.6
miR1857	57.78	42.22	A(9)U(17)G(13)C(6)N(0)	45	−11.8	−0.62
miR2082	52.64	47.36	A(12)U(18)G(19)C(8)N(0)	57	−18.66	−0.66
miR2098	65.52	34.48	A(19)U(38)G(20)C(10)N(0)	87	−21.1	−0.7
miR2105	63.81	36.19	A(30)U(37)G(22)C(16)N(0)	105	−27.7	−0.72
miR2275	47.7	52.3	A(16)U(15)G(16)C(18)N(0)	65	−21.3	−0.62
miR2606	57.5	42.5	A(10)U(13)G(9)C(8)N(0)	40	−10.2	−0.6
miR2628	41.09	58.91	A(21)U(22)G(21)C(9)N(0)	73	−18.6	−0.62
miR2634	74.29	25.71	A(10)U(16)G(5)C(4)N(0)	35	−12.3	−1.36
miR2866	64.95	35.05	A(19)U(43(G(17)C(17)N(1)	97	−21.1	−0.62
miR2919	63.24	36.76	A(21)U(22)G(16)C(9)N(0)	68	−22.2	−0.888
miR393	53.85	46.15	A(11)U(17)G(13)C(11)N(0)	52	−16.2	−0.67
miR396	61.23	38.77	A(10)U(20)G(10)C(9)N(0)	49	−16.5	−0.86
miR397	54.17	45.83	A(8)U(18)G(10)C(12)N(0)	48	−14	−0.63
miR3979	60	40	A(11)U(16)G(8)C(10)N(0)	45	−11.2	−0.62
miR399	57.15	42.85	A(14)U(10)G(10)C(8)N(0)	42	−13.3	−0.73
miR414	49.11	50.89	A(34)U(21)G(29)C(28)N(0)	112	−33.4	−0.6
miR437	58.74	41.26	A(18)U(19)G(16)C(10)N(0)	63	−21.4	−0.82
miR4413	68.43	31.57	A(21)U(31)G(17)C(7)N(0)	76	−14.6	−0.6
miR444	63.83	36.17	A(8)U(22)G(10)C(7)N(0)	47	−10.2	−0.59
miR4995	42.86	57.14	A(13)U(14)G(18)C(18)N(0)	63	−23.7	−0.65
miR5015	54.24	45.76	A(9)U(23)G(15)C(12)N(0)	59	−16.9	−0.625
miR5021	50	50	A(12)U(11)G(14)C(9)N(0)	46	−16.2	−0.7
miR5079	76.93	23.07	A(25)U(35)G(8)C(10)N(0)	78	−11	−0.61
miR5265	67.22	32.78	A(24)U(17)G(11)C(9)N(0)	61	−13.8	−0.69
miR5267	68.75	31.25	A(42)U(46)G(21)C(19)N(0)	128	−37.7	−0.94
miR5338	61.62	38.38	A(90)U(85)G(64)C(45)N(0)	284	−69	−0.63
miR537	71.43	28.57	A(12)U(18)G(10)C(2)N(0)	42	−8.3	−0.69
miR5489	52.31	47.69	A(18)U(16)G(18)C(13)N(0)	65	−20.7	−0.66
miR5542	67.2	32.8	A(36)U(48)G(30)C(11)N(0)	125	−25.2	−0.6
miR5565	39.55	60.45	A(35)U(46)G(33)C(20)N(0)	134	−39.6	−0.74
miR5641	70.28	29.72	A(22)U(30)G(14)C(8)N(0)	74	−21.3	−0.96
miR5658	81.82	18.18	A(14)U(22)G(7)C(1)N(0)	44	−5.8	−0.72
miR5662	51.84	48.16	A(108)U(75)G(91)C(80)N(1)	355	−103.2	−0.6
miR773	53.66	46.34	A(6)U(16)G(12)C(7)N(0)	41	−16.2	−0.85
miR779	57.86	42.14	A(29)U(41)G(33)C(18)N(0)	121	−31.1	−0.609
miR781	68.19	31.81	A(9)U(21)G(9)C(5)N(0)	44	−13.1	−0.93
miR837	54.89	45.11	A(29)U(44)G(30)C(30)N(0)	133	−38.4	−0.63
miR838	49.39	50.61	A(138)U(144)G(150)C(139)N(0)	571	−177.3	−0.613
miR863	63.64	36.36	A(13)U(15)G(9)C(7)N(0)	44	−10.8	−0.67
miR865	76.93	23.07	A(22)U(18)G(4)C(8)N(0)	52	−8.7	−0.72
miR867	81.64	18.36	A(45)U(35)G(9)C(9)N(0)	98	−17.6	−0.9
miR902	68.86	31.14	A(20)U(22)G(11)C(8)N(0)	61	−12.7	−0.66

### 3.3 Sequence Alignment and Phylogenetic Analysis of New miRNA

**FIGURE 3:**
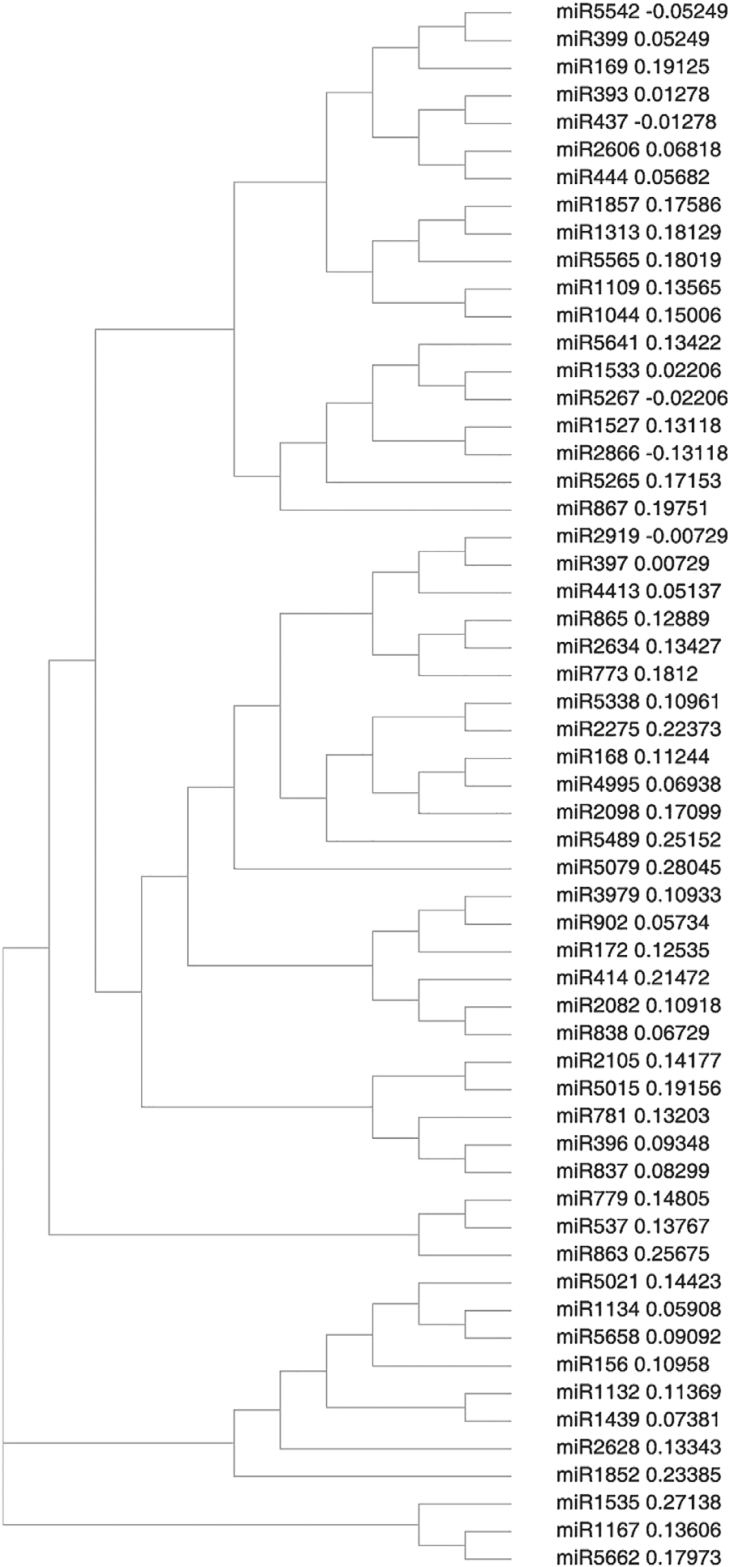
Phylogenetic relationship among predicted miRNA families in cluster bean.

Primary and mature plant miRNAs are highly conserved among distantly related plant species. This high level of conservation between taxa was used as an ancillary criterion for miRNA annotation. Comparison of the precursor sequences of the predicted miRNAs with each other showed that most members could have a high degree of sequence similarity with others. Three different clusters were formed which I cluster was the largest and was divided into two sub-clusters. Sub-cluster I consists of miR5542, miR399, miR169 miR393, miR437, miR2606, miR444, miR1857, miR1313, miR5565, miR1109, miR1044, miR5641, miR1533, miR5267, miR1527, miR2666, miR5265, and miR867. Sub-cluster II consists of miR2919, miR397, miR4413, miR865, miR2634, miR773, miR5338, miR2275, miR168, miR4995, miR2093, miR5489, miR5079, miR3979, miR902, miR172, miR414, miR2082, miR838, miR2105, miR5015, miR781, miR396, and miR837and comprised of largest number of miRNA families as in Figure 3. Cluster II consists of miR5021, miR1134, miR5658, miR156, miR1132, miR1439, miR2628 and miR1852. Cluster III, which is the smallest of all comprises miR1535, miR1167, and miR5662.

### 3.4 Target Identification

The target identification procedure using a computational method is simple and efficient, yielding possible targets that aid in the subsequent study. C-mii’s target prediction menu consists of four submenus: sequence loading, target scanning, miRNA-target folding, and target annotation. Different miRNA targets are represented in Table 2. The acceptable length of an input sequence for target identification was extended to 20,000 nt. In the present study, 2,554 sequences previously reported as miRNA-specific targets from 47 different plant species were uploaded and selected for target scanning. Using the plus strand, 6,164 sequences were identified as miRNA binding sites for 361 miRNA families and 670 members. All of them were selected as input for miRNA target folding at 37°C. About 3,068 sequences were successfully folded and these sequences were used as input for target annotation using Gene Ontology (GO). The target annotation module predicts the function for potential targets. Target identification results are provided in Table 2 with their targeted EST ID. A total of 57 predicted miRNAs from 37 miRNA families were found to regulate 623 target transcripts. miRNAs can potentially regulate many distinct genes, implying that these genes belong to several gene families engaged in various biological, cellular, and molecular processes (Dehury et al., 2013). However, several miRNAs with unclear roles were discovered (miR1104, miR1167, miR1313, miR1852, miR2866, miR2919, miR3522, miR4413, miR5267, miR5338, miR537, miR5489, miR779, miR781, miR867, miR863, miR444, miR5265, and miR5542).

**TABLE 2:** Major potential target genes for predicted miRNAs in cluster bean.

miRNA	Targeted protein	Targeted EST ID	Description
miR156	SPL4, SPL13, SBP2, SBP1, SPL5	EG982824.1 GLL094_E10_036	Squamosa promoter-binding-like protein
	UBC	EG979243.1 GLL062_C07_030	Ubiquitin-conjugating enzyme E2
miR172	2AAG	EG985097.1 GLE041_H10_033	Serine/threonine-protein phosphatase
	ARF	EG990107.1 GLE087_E01_004	ADP-ribosylation factor
miR393	RK20	EG987334.1 GLE062_E02_004	50S ribosomal protein
miR3979	SPZX	EG989678.1 GLE083_C05_022	Serpin-ZX
miR397	G3PA	EG975619.1 GLL029_A09_040	Glyceraldehyde-3-phosphate dehydrogenase A
miR414	AGO1_	EG985306.1 GLE044_A02_008	Protein argonaute
	ALFIN	EG986332.1 GLE052_H02_001	PHD finger protein Alfin1, PHD finger protein ALFIN-LIKE 7
	C3H2	EG982280.1 GLL08_C06_022	Zinc finger CCCH domain-containing
	CD48C	EG983335.1 GLE022_G09_034	Cell division control protein 48 homolog C
	CESA6, CESA5, CESA2, CESA9, CESA9	EG983155.1 GLE021_B02_007	Cellulose synthase A catalytic subunit 5, Cellulose synthase A catalytic subunit 2, Probable cellulose synthase A catalytic subunit 9, Cellulose synthase A catalytic subunit 9
	TGT2	EG986117.1 GLE050_G11_042	Trihelix transcription factor GT-2, Trihelix transcription factor GTL1, Trihelix transcription factor GTL2
miR437	CCMC	EG985481.1 GLE045_F11_043	Putative cytochrome c biosynthesis ccmC-like mitochondrial protein
miR773	P2C	EG988418.1 GLE072_A02_008	Protein phosphatase 2C and cyclic nucleotide-binding/kinase domain-containing protein
miR837	HMDH1	EG988124.1 GLE06_B01_007	3-hydroxy-3-methylglutaryl-coenzyme A reductase 1
	Q8OMT	EG976284.1 GLL034_E07_028	8-hydroxyquercetin 8-O-methyltransferase, Isoflavone-7-O-methyltransferase 9
miR838	NAC	EG989253.1 GLE07_B05_023	NAC domain-containing protein 18, NAC domain-containing protein 68, NAC domain-containing protein 67, NAC domain-containing protein 29, NAC domain-containing protein 48
	HMA4	EG990669.1 GLE091_H04_009	Putative cadmium/zinc-transporting ATPase
miR865	RL	EG988689.1 GLE074_F05_019	60S ribosomal protein
miR902	MNS2	EG989919.1 GLE085_F05_019	Mannosyl-oligosaccharide 1, Mannosyl-oligosaccharide 1
miR1109	AGO4	EG986669.1 GLE056_A10_040	Protein argonaute
miR1132	CML35	EG985964.1 GLE04_C12_046	Probable calcium-binding protein
	RANA1	EG985231.1 GLE043_C03_014	GTP-binding nuclear protein Ran-A1
miR1134	GAST1	EG989127.1 GLE078_H10_033	Gibberellin-regulated protein
miR1439	CML	EG985964.1 GLE04_C12_046	Probable calcium-binding protein
miR1527	CAF	EG981106.1 GLL079_F10_035	Probable CCR4-associated factor 1 homolog 7
miR1533	GALE	EG984760.1 GLE038_G01_002	UDP-glucose 4-epimerase GEPI42, UDP-glucose 4-epimerase, UDP-glucose 4-epimerase 1, UDP-glucose 4-epimerase 2, UDP-glucose 4-epimerase GEPI48
	MT	EG983379.1 GLE011_B12_047	Metallothionein like protein
	JKD_ MGP,NUC	EG990549.1 GLE090_G04_010	Zinc finger protein JACKDAW, Zinc finger protein MAGPIE, Zinc finger protein NUTCRACKER
miR1857	GALE	GO542112.1 Mdfrjg3507I06.g1 Apple_E	UDP-glucose 4-epimerase GEPI48, UDP-glucose 4-epimerase 2, UDP-glucose 4-epimerase 3, UDP-glucose 4-epimerase GEPI42, UDP-glucose 4-epimerase 1
miR2098	RS193	EG981936.1 GLL086_H09_033	40S ribosomal protein
miR2105	REV, HOX9, HOX9, HOX10, HOX10	EG990607.1 GLE091_C08_030	Homeobox-leucine zipper protein
miR2275	HSP81 82 83 90-1	EG986829.1 GLE057_F03_011	Heat shock protein 81-1, Heat shock protein 81-3, Heat shock protein 81-2, Heat shock protein 90-1
miR2628	DXS	EG980801.1 GLL076_E12_044	Probable 1-deoxy-D-xylulose-5-phosphate synthase
miR2606	TSJT1	EG986569.1 GLE055_B02_007	Stem-specific protein TSJT1
miR2634	AG	EG990361.1 GLE089_H09_033	Floral homeotic protein AGAMOUS
	GAOX2	EG987827.1 GLE067_C01_006	Gibberellin 20 oxidase 2,
miR4995	RLA	EG986778.1 GLE057_B04_015	60S acidic ribosomal protein
miR5021	2AAB_	EG986207.1 GLE051_F09_035	Serine/threonine-protein phosphatase 2A
	3MAT 5MAT1,AGCT	EG983947.1 GLE030_E09_036	Malonyl-coenzyme A:anthocyanin 3-O-glucoside-6″-O-malonyltransferase
	AGL11, AGL5, AGL1_,MAD21	EG975055.1 GLL011_C02_006	Agamous-like MADS-box protein, MADS-box transcription factor 21
	ARI7,ARI8,ARI6_	EG987656.1 GLE065_E09_036	Probable E3 ubiquitin-protein ligase
	ASP	EG984370.1 GLE035_A02_008	Aspartic proteinase Asp1
	BGLS	EG983200.1 GLE021_E07_028	Non-cyanogenic beta-glucosidase,Cyanogenic beta-glucosidase
miR5079	EXP13	EG985350.1 GLE044_D07_029	Expansin-A13
miR5565	ADLO1	EG986858.1 GLE057_H05_017	Protein ARABIDILLO 1
miR5641	CAX3	EG984929.1 GLE040_C12_046	Vacuolar cation/proton exchanger
miR5658	AGL11	EG975055.1 GLL011_C02_006	Agamous-like MADS-box protein AGL11
	CSLG2	EG976517.1 GLL036_G04_010	Cellulose synthase-like protein G2, Cellulose synthase-like protein G1, Cellulose synthase-like protein G3
	GASA4	EG987384.1 GLE062_H11_041	Gibberellin-regulated protein 4
	GLYT3	EG975650.1 GLL029_D02_005	Probable glycosyltransferase
	HSFA9, HSF30, HFA2C,HFA6B	EG985970.1 GLE04_D05_021	Heat stress transcription factor A-9, Heat stress transcription factor A-2e, Heat shock factor protein HSF30, Heat stress transcription factor A-2c, Heat stress transcription factor A-6b
miR168	AGO1	EG985306.1 GLE044_A02_008	Protein argonaute 1
	DHSA	EG985277.1 GLE013_C07_030	Succinate dehydrogenase [ubiquinone] flavoprotein subunit
miR169	AL2B7	EG983762.1 GLE029_F12_043	Aldehyde dehydrogenase family 2 member B7
miR399	CYSK	EG985107.1 GLE042_A07_032	Cysteine synthase
miR2082	TBB	EG984572.1 GLE036_H07_025	Tubulin beta-6 chain, Tubulin beta-3 chain, Tubulin beta-7 chain, Tubulin beta-4 chain
miR5662	PIP	EG976742.1 GLL013_A03_016	Probable aquaporin PIP1-2, AquaporinPIP1-2, Aquaporin PIP1-3/PIP1-4, Aquaporin PIP1-3, Aquaporin PIP1-1
miR1535	C3H11,C3H21	EG979135.1 GLL061_C02_006	Zinc finger CCCH domain-containing protein 11, Zinc finger CCCH domain-containing protein 21

miRNA414, miR5658, and miR5021 have the highest number of targets. Some miRNAs such as miR156, miR172, miR414, miR1533, and miR5021 had more than one target while some miRNA families have common targets as represented in Table 3.

**TABLE 3:** Common targets of predicted miRNA families in cluster bean

miRNA	Common target	Traits associated	Function
miR172, miR5021	PP2A	Biotic and abiotic stress	Serine/threonine protein phosphatase
miR5021, miR838	FRI	Iron content	Ferritin
miR156, miR1533, miR5021	UBC	Pattern triggered immunity	Ubiquitin-conjugating enzyme
miR837,miR838, miR5021	TRL	Role in plant tolerance of oxidative stress	Thioredoxin
miR5566, miR156	TBG	Plant growth and morphogenesis	Tubulin
miR5021 ,miR5658	PATL	Cell formation	Patellin
miR1533, miR838	P2C	Stress and developmental process	Probable protein phosphatase
miR5021, miR5658, miR396	MADS	Flower development	Floral homeotic protein

**FIGURE 4:**
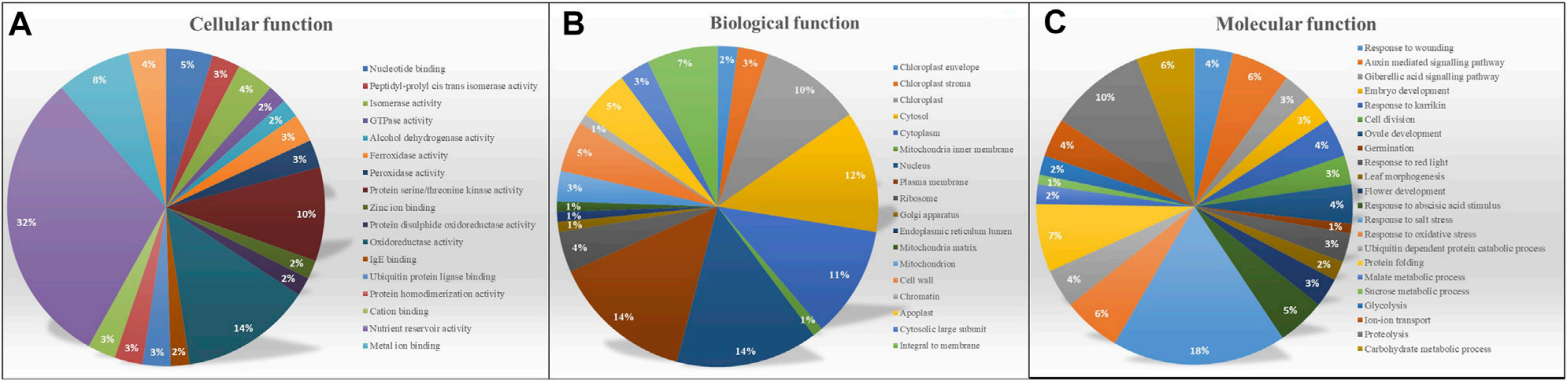
GO annotation for output targets and their distribution in three categories **(A)** Molecular function **(B)** Biological process **(C)** Cellular component.

To gain a better understanding of the functional characteristics of these miRNA targets, the GO annotations of all projected targets were examined. All targets were divided into three GO categories based on the ontological meanings of the GO words, as shown in Figures 4A–C molecular function, biological process, and cellular component. Predicted targets in the molecular function category were related to nutrition reservoir activity (32%), followed by nucleotide binding and protein serine/threonine kinase activity. The bulk of the targets in the biological process was potentially implicated in cellular activities and metabolic processes involving salt stress (18%) and proteolysis (10%). Other genes were found to be involved in a variety of signaling pathways, including the gibberellic acid signaling pathway, auxin-mediated signaling, and abscisic acid stimulation. Target genes in the cellular component category were assigned to the subcategories cytoplasm (24%) chloroplast (15%) nucleus (14%) mitochondria and membrane. Major GO IDs were selected based on their function and classified into biotic and abiotic, and carbohydrate metabolism-related categories as represented in Supplementary Table S5.

The Gene Ontology provides the logical structure of the biological functions and their relationships to one another, manifested as a directed acyclic graph.

1) GO: 0009615 is involved in any process that results from a stimulus of the virus.This GO ID was found to be associated with other GO IDs such as GO:0008150, GO:0050896, GO:0009605, GO:0009606, GO:0043207, GO:0051704, GO:51707.
2) GO: 0009414 relates to a change in the activity of cells as a result of water deprivation. This GO ID was found to be interconnected with GO:0008150, GO:0050896, GO:0042221, GO: 0001101, GO:0010035, GO:0006950, GO:0009415, GO:1901700, and GO:00009628.
3) GO:0005985 relates to all the chemical reactions and pathways involving sucrose, the disaccharide fructofuranosyl-glucopyranoside. This GO ID was associated with GO: 0008150, GO:0005984, GO:0044262, GO:0009311, GO:0005975, GO:0044238, GO:0044237, GO:0009987, GO:0008152, GO:0071704, and GO:008152.

### 3.5 Gene Regulatory Networks of Cluster Bean Target Genes in GENEMANIA

**FIGURE 5:**
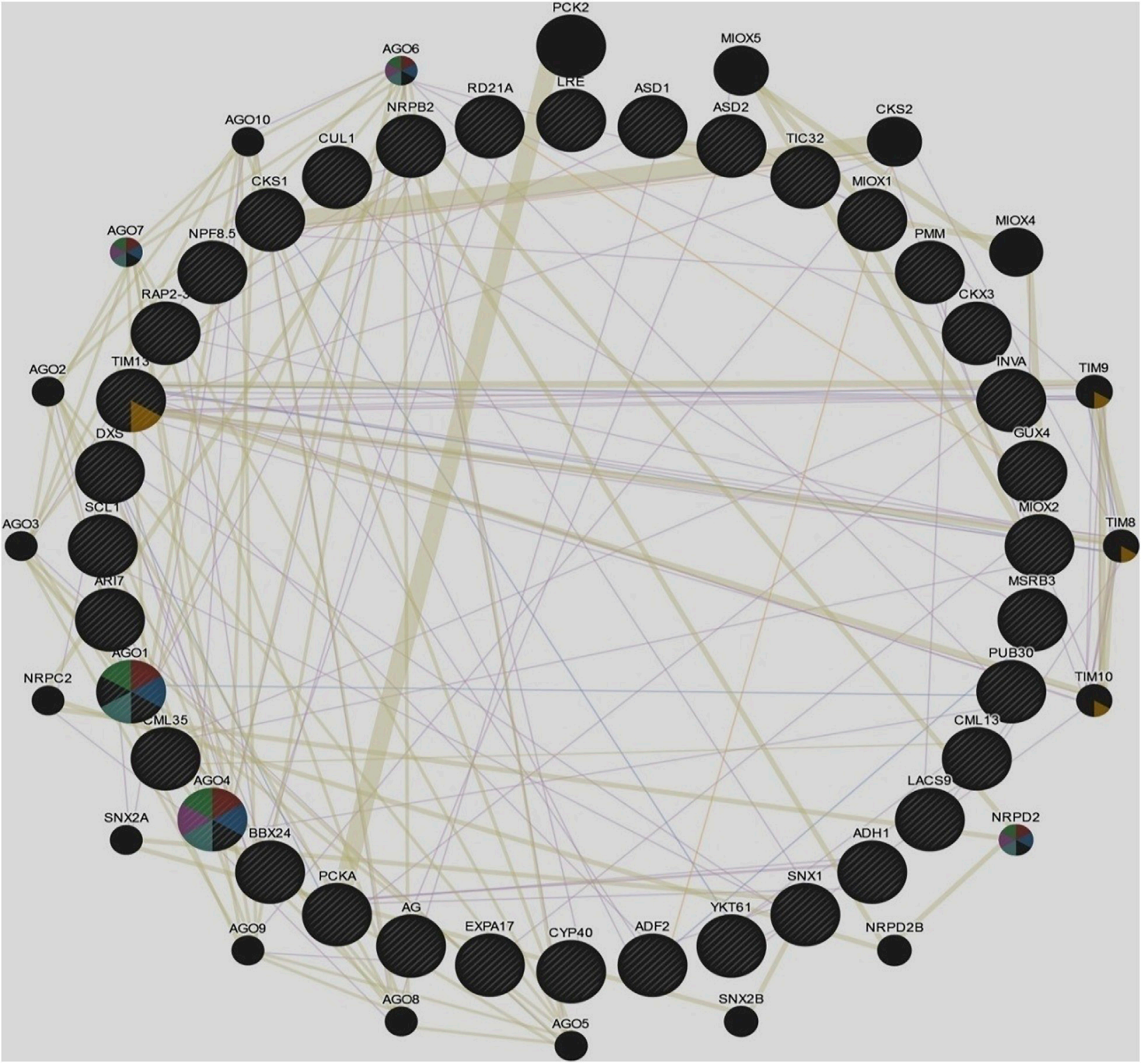
Gene regulatory networks of cluster bean target genes in GENEMANIA against *Arabidopsis thaliana*.

Target genes of predicted miRNA families of cluster bean were used to make a regulatory network in GENEMANIA and to predict the function of these genes (Figure 5). Following genes were found and a regulatory network was predicted against *Arabidopsis thaliana*. The genes were CML 13, MIOX1, SCL1, RD21A, AGO1, GUX4, INVA, TIM13, ADH1, LACS9, CYP40, MIOX2, AGO4, CKS1, AR17, CML35, PMM, ASD1, ADF2, PUB30, CUL1, MSRB3, DXS, AG, TIC32, LRE, PCKA, SNX1, ASD2, CKX3, YKT61, STO, EBP, EXP17 and RPB2. The network represents the involvement of various additional genes and their function (Supplementary Table S6) using STRING.

Various genes comprised of different networks such as co-expression (55.01%), shared protein domain (35.32%), predicted (6.35%), and co-localization (3.32%). The given network indicates the involvement of various genes functioning in mainly RNA interference, post-transcriptional gene silencing by RNA, the establishment of protein localization to mitochondrion, and production of siRNA involved in RNA interference.

### 3.6 Identification of Transcription Factors and Depicting Regulatory Network Model Between miRNA and Transcription Factor

Many of the predicted targets were annotated to be transcription factors. To find some correlation between miRNA and transcription factors, the plant transcription factor database was used to identify transcription factors from the input sequences. EST sequences were used as input sequences and ESTScan 3.0 was employed to identify CDS regions of input nucleic acid sequences which translated them to protein sequences. By checking “Best hit in *Arabidopsis thaliana*,” the transcription factors such as B3 family protein, basic pentacysteine 6 and 4, bHLH family protein, basic leucine-zipper 70, TGACG motif-binding factor 6, C3H family protein, MIKC_MADS family protein, nuclear factor Y, and TCP family protein etc. were identified in EST database (Supplementary Table S7).

**FIGURE 6:**
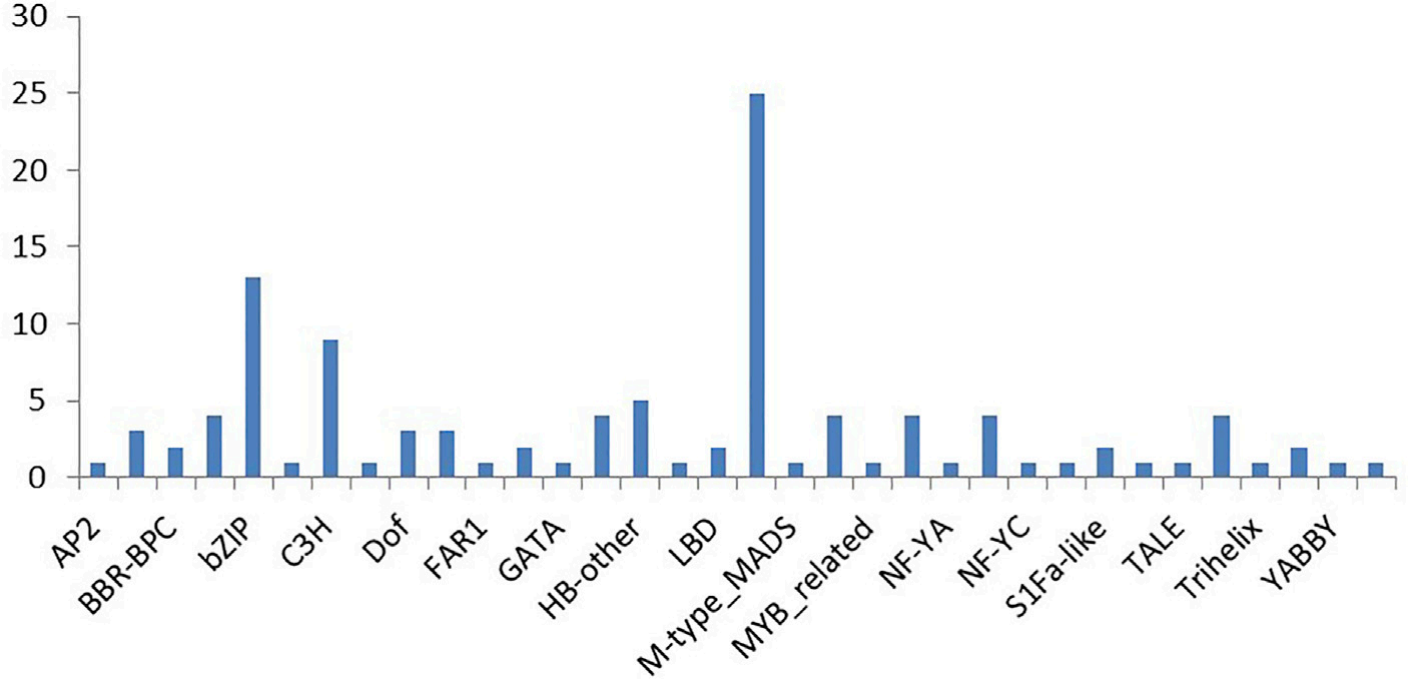
Major transcription factor classes identified from cluster bean ESTs.

**FIGURE 7:**
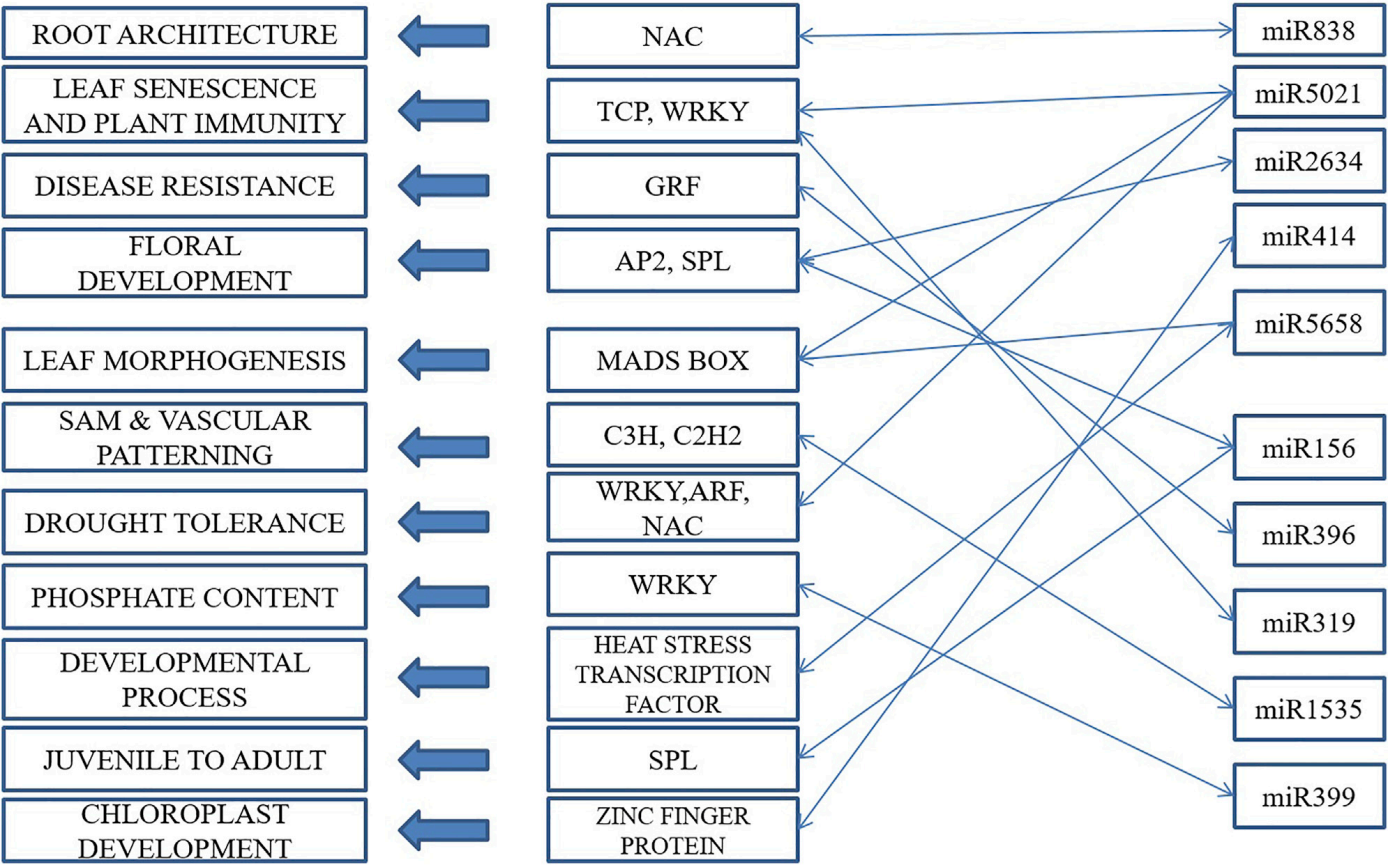
A proposed model of interactions among transcription factors and miRNAs for gene regulation in cluster bean.

This led to the identification of many transcription factors, the bulk of which belonged to the MIKC MADS family protein basic leucine-zipper and the C3H family protein (Figure 6). A model is well represented in Figure 7, showing the interaction between miRNA and transcription factor genes, as well as their impact on many developmental processes and metabolic activities in plants. miRNAs and TFs predicted from the EST database enable combinatorial gene regulation with varied roles that can be used to enhance crops. The combination of miRNA and their targets, which are mostly transcription factors, resulted in the construction of a model suggesting a clear-cut link that leads to the regulation of many developmental and metabolic processes. MiR156, for example, interacts with AP2 and SPL to govern floral growth as well as the juvenile-to-adult transition. Similarly, miR5021 and miR5658 target MADS BOX, which is known to play a crucial role in leaf and flower growth. Furthermore, miR5658 targets heat stress transcription factors and is involved in drought stress and developmental processes such as growth and reproduction. miR838 targets NAC, which is essential for root growth.

## 4 Discussion

In recent times numerous studies carried out by various researchers have demonstrated that plant miRNAs are involved in various biological and developmental processes and physiological responses. Gaining insights into miRNAs and their targets help us to understand the array of biological events underlying miRNA-mediated regulation and will further elucidate the functional importance of miRNAs (Pani and Mahapatra, 2013; Chaudhary, 2019). A substantial number of miRNAs are conserved among the plant species and it has been observed plant miRNAs and their target sites bind to each other with perfect or near to perfect complementarity (Wang et al., 2004; Schwab et al., 2005; Patanun et al., 2013). This provides an opportunity for identification of miRNAs and their targets in different crop plants by comparing/aligning with the known miRNA sequences. This approach has been widely used to identify miRNA and their targets in various crop plants (Sunkar and Jagadeeswaran, 2008; Dehury et al., 2013; Gasparis et al., 2017; Vivek, 2018). Here, we report the identification of miRNAs and their potential targets in cluster bean by mining EST database.

Cluster bean is an emerging economically important legume with wide range of industrial applications (Rajaprakasam et al., 2021). Despite, high industrial importance of the guar gum this crop is considered as orphan as little progress has been made and availability of genetic and genomic resources is very limited in this crop (Ravelombola et al., 2021). Recently, advanced molecular and genomic approaches have been utilized in this orphan crop to combat the yield losses and elucidate the function of galactomannan related genes (Al-Qurainy et al., 2019; Kumar et al., 2020; Rajaprakasam et al., 2021; Grigoreva et al., 2021a; Grigoreva et al., 2021b; Acharya et al., 2022). Attempts have also been made to sequence cluster bean genome (Grigoreva et al., 2019); however, this first sequencing attempt has a gap of 50% (Grigoreva et al., 2019).

Recent studies are now gradually overcoming the limitations, however, still limited information is available on miRNA-mediated post-transcriptional regulation of metabolic pathways in cluster bean. In few reports, deep sequencing has been applied to identify miRNAs in cluster bean (Sahu et al., 2018; Tyagi et al., 2018). Deep sequencing is the most commonly employed technique for miRNA identification in plant species whose whole genome sequence is not known (Peláez et al., 2012; Tyagi et al., 2018; Tiwari et al., 2021). However, this technique generates huge amount of data and needs expertise for data analysis. Moreover, next-generation sequencing (NGS) is costly and not possible for routine applications (Gasparis et al., 2017). This limitation can be overcome by using *in silico* approaches that have emerged as fast and cheap alternative and uses databases like EST and GSS (Vivek, 2018).

Similar strategy was applied in this study and EST database was used to mine miRNAs using computational approach. A large number of miRNA families were identified in cluster bean (Table 4) belonging to drought, cold, biotic stress tolerance, and salt-specific stress conditions. As previously reported in other species, the majority of the cluster bean precursor miRNA sequences varied in length from 35 nt to 592 nt with an average value of 100 (Wang et al., 2012; Singh et al., 2016). The length of the mature miRNAs was found to be 20–22 nt long. Similar length of mature miRNAs was reported in earlier miRNA identification studies in soybean, and switchgrass (Xie et al., 2010; Li et al., 2015). The nucleotide composition showed that at the first position at 5′ end of mature miRNA uracil was pre-dominant, similar to observations made by Zhang et al. (2006). It was observed that the average AU content (61.18%) was higher than the GC content (38.18). The higher AU content depicts a comparatively less stable pre-miRNA secondary structure that is easily recognized by RISC complex and converted into mature miRNA. The minimal folding free energy (MFE) is an important determining factor for the stability of the secondary structure. The lower the values of MFE, the higher the thermodynamic stability of the secondary structure (Prabu and Mandal, 2010). In the present study, the MFE value was found to be 25.4 (−kcal/mol). The MFE reported in this study was significantly lower than MFE value of earlier reported miRNAs in cluster bean, where the most stable miRNA, cte_miR1134 has MFE of −72.6 kcal/ mol (Sahu et al., 2018). Similarly, the MFE of miRNA families reported by Tyagi et al., 2018 in different tissue-specific miRNAs of cluster bean was quite higher. The MFE values of miRNAs reported in other legumes like peanut (−50.01 kcal/mol; Chi et al., 2011), French bean (-35.0 to -51.2 kcal/mol; Peláez et al., 2012), chickpea (−50.1419 kcal/mol; Kohli et al., 2014), and lentil (-44.07 kcal/mol; Hosseini et al., 2021). Nowadays, to differentiate miRNA from other RNAs an MFEI value of higher than 0.85 is a potential criterion (Wang et al., 2012). The cluster bean pre-miRNAs identified in the present investigation showed MFEI values ranging from 0.59 to 1.39 (−kcal/mol) with an average value of 0.70 (−kcal/mol). The MFEI values reported in this study were similar to that of wheat (0.79–1.85−kcal/mol; Gasparis et al., 2017) and lentil (−1.02 kcal/mol; Hosseini et al., 2021).

**TABLE 4:** List of predicted miRNA families in cluster bean.

Input sequence Id	Precurson start/Stop	Known mature miRNA sequence	Predicted miRNA sequence	Predicted miRNA family	Homolog miRNA
EG990325.1 GLE089_F01_003	554–644	5′:UGA​CAG​AAG​AAA​GAG​AGC​AC:3′	5′:UGA​GAG​AAG​AAA​GAG​AGG​AA:3′	miR156	ath-miR156h
EG980529.1 GLL073_G12_042	382–429	5′:UCG​CUU​GGG​CAG​AUC​GGG​AC:3′	5′:UCG​CUU​GGU​CAG​AUC​UGC​GC:3′	miR168	sof-miR168b
EG984942.1 GLE040_D12_045	201–258	5′:GAG​CCA​AGG​AUG​ACU​UGC​CGU:3′	5′:GAG​CCA​AGG​GUG​ACU​UAA​UGU:3′	miR169	vvi-miR169l
EG985097.1 GLE041_H10_033	324–379	5′:AGA​AUC​UUG​AUG​AUG​CUG​CAU:3′	5′:AGA​AUC​UUG​AUC​AUC​CUG​UGU:3′	miR172	gma-miR172b
EG987334.1 GLE062_E02_004	369–420	5′:UCC​AAA​GGG​AUC​GCA​UUG​AUC:3′	5′:UCC​AAA​GGG​AUC​GCA​UGU​CAC:3′	miR393	gma-miR393
EG978583.1 GLL055_H01_001	205–253	5′:UUC​CAC​AGC​UUU​CUU​GAA​CUU:3′	5′:UUU​CAC​AGC​UUU​CUU​GAU​UGU:3′	miR396	osa-miR396c
EG975619.1 GLL029_A09_040	413–460	5′:UCA​UUG​AGU​GCA​GCG​UUG​ACG:3′	5′:UCU​CUG​AAU​GCA​GCG​UUG​ACU:3′	miR397	pab-miR397
EG990966.1 GLE094_G01_002	207–248	5′:UGC​CAA​AGG​AGA​AUU​GCC​C:3′	5′:AUC​CAA​AGG​UGA​AUU​GCU​C:3′	miR399	tae-miR399
EG985485.1 GLE045_G03_010	317–428	5′:UCA​UCC​UCA​UCA​UCA​UCG​UCC:3′	5′:UUA​UCA​UCA​UCC​UCA​UCA​UCC:3′	miR414	osa-miR414
EG978745.1 GLL057_D04_013	137–199	5′:AAA​GUU​AGA​GAA​GUU​UGA​CUU:3′	5′:AUA​GCU​AGA​GAA​GUU​UGA​AAU:3′	miR437	osa-miR437
EG976745.1 GLL038_H10_033	311–357	5′:UGC​AGU​UGC​UGC​CUC​AAG​CUU:3′	5′:UUC​AUU​UGA​AGC​CUC​AAG​CUU:3′	miR444	osa-miR444a.2
EG978435.1 GLL054_D09_037	223–300	5′:UUG​AGG​UGU​UUC​UAC​AGG​CUA:3′	5′:UUG​AGG​AGU​UUC​UAC​AUU​CAA:3′	miR537	ppt-miR537a
EG989794.1 GLE084_D07_029	632–672	5′:UUU​GCU​UCC​AGC​UUU​UGU​CUC:3′	5′:UUU​GCU​UCC​AGC​UUU​GGU​UUG:3′	miR773	ath-miR773
EG985501.1 GLE045_H05_017	388–541	5′:UUG​UAG​UGC​AUA​UUU​GUU​UU:3′	5′:UUG​UUA​UGC​AUG​UUG​GUU​UU:3′	miR1044	ppt-miR1044
EG989165.1 GLE017_B06_023	380–512	5′:UAG​UGG​GAG​AUU​UUG​UGU​AAC:3′	5′:UAU​UGG​GAA​AUU​UUG​UGU​CGC:3′	miR1109	smo-miR1109
EG974827.1 GLL020_C10_038	335–369	5′:CAU​UAU​GGA​ACG​GAA​GGA​G:3′	5′:CAU​UAU​GGA​AAG​GAA​GGG​A:3′	miR1132	tae-miR1132
EG986974.1 GLE05_A06_024	36–91	5′:CAA​CAA​CAA​CAA​GAA​GAA​GAA​GAU:3′	5′:GAA​CAA​GAA​GAA​GAA​GAA​GAA​GAA:3′	miR1134	tae-miR1134
EG975778.1 GLL02_E10_036	44–89	5′:GGG​GUG​UGA​UGA​UUU​GAA​AC:3′	5′:UGG​GUG​UGA​GGA​UUU​GAG​AC:3′	miR1167	cre-miR1167
EG989769.1 GLE084_B06_023	158–251	5′:UAC​CAC​UGA​AAU​UAU​UGU​UCG:3′	5′:UUC​AAU​UGA​AAU​UAU​UGG​UCG:3′	miR1313	pta-miR1313
EG989141.1 GLE079_A10_040	161–752	5′:UUU​UGG​AAC​GGA​GUG​AGU​AUU:3′	5′:UUA​UGA​AAC​GGA​GUG​AGU​AAU:3′	miR1439	osa-miR1439
EG981106.1 GLL079_F10_035	51–96	5′:UAA​CUC​AAC​CUU​ACA​AAA​CC:3′	5′:GAA​CUC​AAG​CUA​ACA​AAA​CC:3′	miR1527	gma-miR1527
EG975750.1 GLL02_C09_038	453–529	5′:AUA​AUA​AAA​AUA​AUA​AUG​A:3′	5′:UUA​AUA​AAA​AUU​AUA​AAA​A:3′	miR1533	gma-miR1533
EG981151.1 GLL010_F06_019	47–191	5′:CUU​GUU​UGU​GGU​GAU​GUC​U:3′	5′:CUG​GUC​UGU​GGU​GCU​GUC​C:3′	miR1535	gma-miR1535
EG983098.1 GLL01_H03_009	96–177	5′:AUA​UGG​AUU​CAG​AAU​GCA​GGU:3′	5′:AUA​AGG​AAA​CAG​AAU​GCA​GGA:3′	miR1852	osa-miR1852
EG975952.1 GLL031_C04_014	116–160	5′:UGG​UUU​UUU​UGG​AGC​AUG​AGG:3′	5′:GGA​AUU​UUU​UGG​AGC​AGG​AGG:3′	miR1857	osa-miR1857
EG990914.1 GLE094_C01_006	644–700	5′:UGU​GUG​UUC​CGC​UUC​UUC​UUU:3′	5′:AGC​GUC​UUC​UGC​UUC​UUC​UUU:3′	miR2082	ppt-miR2082
EG983237.1 GLE021_H04_009	192–278	5′:CGG​UUU​GUC​AAG​CGG​AGU​GC:3′	5′:UGC​UUU​GUC​AAG​GGG​AGU​GU:3′	miR2098	osa-miR2098
EG978398.1 GLL054_A10_040	393–497	5′:UUG​UGA​UGU​GAA​UGA​UUC​AU:3′	5′:GGG​UGA​UGU​GAU​UGA​UUC​CU:3′	miR2105	osa-miR2105
EG987207.1 GLE061_C06_022	253–317	5′:AGG​AUU​AGA​GGG​ACU​UGA​ACC:3′	5′:ACG​CUU​CGA​GGG​ACU​UGA​AUC:3′	miR2275	zma-miR2275c
EG986569.1 GLE055_B02_007	573–612	5′:UAC​AAU​UCC​UUA​GGU​GCU​UUU:3′	5′:UUC​AGC​UCC​UUA​GGU​GCA​UUU:3′	miR2606	mtr-miR2606a
EG980814.1 GLL076_F12_043	210–282	5′:CAU​GAA​AGA​AUG​AUG​AGU​AA:3′	5′:CAG​GUG​AGA​AUG​AUG​AGG​AA:3′	miR2628	mtr-miR2628
EG983630.1 GLE027_H07_025	570–604	5′:UUU​AUU​CUC​AGU​UUG​UUG​CUC:3′	5′:UUU​AUU​CUC​AGU​UUU​UUG​AUC:3′	miR2634	mtr-miR2634
EG982808.1 GLL094_D08_029	586–682	5′:UCU​AGU​UUG​UGU​UCA​GCA​UC:3′	5′:UCU​AAU​UUG​UGU​UCA​CUU​UC:3′	miR2866	osa-miR2866
EG988391.1 GLE071_F12_043	404–471	5′:AAG​GGG​GGG​GGG​GGA​AAG​A:3′	5′:AGG​GGG​AGG​GGG​GGA​AAU​U:3′	miR2919	osa-miR2919
EG988087.1 GLE016_B04_015	180–224	5′:UCU​CUC​UCU​CCC​UUG​AAG​GC:3′	5′:UCA​CUC​UCU​CCC​UUG​CAG​UU:3′	miR3979	osa-miR3979
EG976112.1 GLL032_G08_026	113–188	5′:UAA​GAG​AAU​UGU​AAG​UCA​CU:3′	5′:UCA​CUG​AAU​UGU​AAG​UUA​CU:3′	miR4413	gma-miR4413b
EG986778.1 GLE057_B04_015	648–710	5′:AGG​CAG​UGG​CUU​GGU​UAA​GGG:3′	5′:AAG​CAG​UGG​CUU​GGU​CAA​GGC:3′	miR4995	gma-miR4995
EG986237.1 GLE051_H12_041	18–76	5′:UCU​GUU​GUU​GUU​GGU​GUU​AUG:3′	5′:UCU​GUU​GUU​GUU​GUU​GUU​GUU:3′	miR5015	ath-miR5015b
EG985015.1 GLE041_B08_031	5–586	5′:UGA​GAA​GAA​GAA​GAA​GAA​AA:3′	5′:GGA​GAA​GAA​GAG​AAA​GGA​AA:3′	miR5021	ath-miR5021
EG990900.1 GLE094_B02_007	644–721	5′:UUU​GGA​UCU​GUU​AUU​UUG​GUA​U:3′	5′:UUU​CUU​UCU​GUU​AUU​UUG​GAA​U:3′	miR5079	osa-miR5079
EG982261.1 GLL08_B02_007	154–214	5′:AAG​UGA​UGU​UGG​AAU​GGU​UA:3′	5′:UAG​UGA​AGU​UGG​AAU​AAU​UA:3′	miR5265	mtr-miR5265
EG981105.1 GLL079_F09_035	141–268	5′:AGG​CAU​UUG​CUA​GAA​UAC​ACC​CAC:3′	5′:AAG​GAU​UUG​CUA​AUA​UAC​ACC​CAC:3′	miR5267	mtr-miR5267a
EG987890.1 GLE067_G12_042	22–305	5′:UGA​AGC​UUC​AGU​UGG​UUG​UAU:3′	5′:AGA​AGC​UUC​AGU​UGG​UUU​UGA:3′	miR5338	osa-miR5338
EG989431.1 GLE017_D06_021	177–241	5′:CAG​GUG​UUC​UCG​AUG​GCU​UCC:3′	5′:CUA​GUG​AUU​UCG​AUG​GCU​UCC:3′	miR5489	osa-miR5489
EG977779.1 GLL049_B04_015	307–431	5′:UUU​GAG​AAG​GUA​UCA​UGA​GAU:3′	5′:UAU​GAG​AAU​GUA​UUA​UGA​GAU:3′	miR5542	osa-miR5542
EG986048.1 GLE050_B05_023	40–173	5′:UUG​UUU​GGA​UGU​UGU​CGG​A:3′	5′:UUG​UUU​GGA​UGC​UGA​UGG​U:3′	miR5565	sbi-miR5565e
EG983888.1 GLE02_H11_041	113–186	5′:UGG​AAG​AAG​AUG​AUA​GAA​UUA:3′	5′:UGG​AAG​AAG​AUG​UGA​AAA​UUA:3′	miR5641	ath-miR5641
EG985672.1 GLE047_E04_012	293–618	5′:AUG​AUG​AUG​AUG​AUG​AUG​AAA:3′	5′:AUG​AUG​AUG​AAG​AAG​AAG​AAG:3′	miR5658	ath-miR5658
EG984029.1 GLE031_E11_044	110–464	5′:AGA​GGU​GAC​CAU​UGG​AGA​UG:3′	5′:AGG​GGU​GAC​CGU​UGG​AGA​CU:3′	miR5662	ath-miR5662

Cluster bean miRNAs were found to be involved in plant development, defense mechanisms, and signal transduction. The projected miRNAs’ functional significance can be easily appreciated by obtaining more information about their target genes. Serine/threonine-protein kinase and ubiquitin carrier protein were anticipated to be the targets of miR172, miR5021, miR156, and miR1533, respectively. Previous reports indicate that ubiquitin carrier proteins plays a significant role in the regulation of various physiological processes such as the recycling of aberrant proteins, metabolic regulation, cell cycle control, and transcription factor (TF) activation (Dreher and Callis, 2007). Serine/threonine-protein kinase plays an important function in signal transduction pathways that contributes to plant defense under both biotic and abiotic stresses (País et al., 2009). It has been reported that miRNAs target TFs, signal transduction factors and metabolic transporters (You et al., 2020).

Cluster bean network analysis of projected miRNAs and their target genes revealed information about the co-regulation of numerous target genes. TFs and miRNAs are both thought to be important regulators of transcriptional events and ultimately, the plant metabolic process (Jangra et al., 2018). TFs regulate gene transcription in the promoter area of the gene, whereas miRNAs regulate gene post transcription in the 3′-untranslated region of the gene, and their respective targets form interconnected functional networks that are critical for the execution of any metabolic activity. Annotation of miRNA-targeted unigenes revealed enrichment of a total 39 TFs such as bHLH (250), Myb (150), Myb related (126), ERF (130), bZip and C2H2 (107), WRKY (100) and NAC (99) (Tyagi et al., 2018). A total of 35 novel TFs were identified which belonged to MIKC_MADS family protein, basic leucine-zipper, and C3H family protein, NAC and WRKY classes. Plant MADS-box genes have been identified as regulators of floral organ identity to control various developmental processes such as the determination of meristem identity of vegetative, inflorescence and floral meristems, root growth, ovule and female gametophyte development, flowering time, fruit ripening and dehiscence (Masiero et al., 2011; Castelán-Muñoz et al., 2019). Certain miRNA-targeted TFs such as NAC and ZF-TFs, implicate their involvement in cellulose synthase-like proteins which is a key factor controlling galactomannan synthesis. The presence of such TFs among the identified miRNA further proves their role in galactomannan pathway. The present study demonstrates the information of miR 5658 family targeting CSL G1, G2, G3 proteins in cluster bean. In both the primary and secondary walls of Arabidopsis, CSL genes are responsible for the majority of glucomannan production (Goubet et al., 2009; Gigli-Bisceglia et al., 2020). In plants, miRNAs function as a negative regulator at the post-transcriptional gene level, either facilitating the cleavage of target transcripts or suppressing their translation, resulting in significant changes in metabolic pathway activity. These are known to regulate cell signaling, oxidative stress, abiotic and biotic stress response, and the development of different tissues, including leaf, stem, anther, root, and flower. Cleavage of the target transcript appears to be the most important and common method of gene control by miRNAs in plants (Catalanotto et al., 2016). The *in silico* identification of predicted miRNAs and their target genes from the EST database suggests a regulatory role in developmental stages. The EST database used in the present study is derived from early and late seed development-specific cDNA libraries. It is the time when galactomannan is synthesized and deposited in seed as a food reserve. Since TFs control gene expression, understanding their function will involve detailed genome-wide functional analysis. Nonetheless, the study provides preliminary information on miRNAs and TFs interplay in cluster bean. *In silico* data analysis gives information regarding miRNA identification and their interaction with TFs and target prediction which can be further supported by wet lab. experiment. We have identified miRNA- SSRs and observed DNA polymorphism in cluster bean germplasm (96 genotypes including commercial varieties and wild species). An amplicon of 450 bp and 120 bp specific to miR169 was observed in cluster bean varieties HG 2-20 and RGC 471, respectively. HG 2-20 is tolerant against bacterial leaf blight, alternaria blight, and root rot, while RGC 471 is tolerant to bacterial blight. The miR169 has been found to be associated with biotic stress tolerance (Song et al., 2018; Šečić et al., 2021). The appearance of amplicon specific to miR169 in these varieties clearly demonstrated their role in disease resistance in cluster bean (Unpublished data).

The miRNAs identified in this study are involved in regulating several genes and further gene expression studies are needed which will aid in the discovery of a new dimension of the miRNA regulatory network during plant growth and development in cluster bean.

## 5 Conclusion

Using ESTs from the NCBI database, 57 putative miRNA families were identified in cluster bean, with MFEI ranging from 0.59 to 1.36 (kcal/mol). A total of 623 target genes were predicted for 57 possible miRNA families, and their expression was suppressed either by cleavage or translational repression. Many of them were implicated in various metabolic processes. Furthermore, miRNA target prediction found that most of these genes are classified as transcription factors. A functional study of the targets revealed that they were engaged in the process of plant development. Most of the anticipated targets were shown to be regulated by several miRNAs, according to network analysis. This study provides an important view of conserved and novel miRNAs, their precursors and targets, and associated TFs. This will help the researchers working on cluster bean miRNAs in unraveling the complex gene regulatory molecular networks. The miRNs identified through *in silico* approach need further experimental validation.

## Data Availability

The original contributions presented in the study are included in the article/Supplementary Material, further inquiries can be directed to the corresponding author.
