# Supplementary material for: In silico Identification of miRNAs and Their Targets in Cluster Bean for Their Role in Development and Physiological Responses
Source: Front Genet. 2022 Jun 30;13:930113. doi: 10.3389/fgene.2022.930113 (PMC9280363; doi:10.3389/fgene.2022.930113)
Supplement: Supplementary file 1 [file DataSheet1.doc]

**Supplementary information**

**Supplementary Table 1:** MiRNA identification steps and number of EST derived sequences in C-mii program

| **MiRNA identification steps** | **Number of sequences at each step** |
| --- | --- |
| Sequence loading | 16503 |
| Homolog search | 9278 |
| Primary miRNA folding | 3068 |
| Precursor miRNA folding | 1167 |

**Supplementary Table 2: Predicted functions of EST Ids in which miRNA families are present against cDNA library of cluster bean**

| **Input sequence** | **Description** | **Function** |
| --- | --- | --- |
| GLE045_H05_017 | Structural constituent of ribosome [*Arabidopsis thaliana* | Translation |
| GLL02_E10_036 | LEXYL1 [*Lycopersicon esculentum*] | Carbohydrate |
| GLL02_C09_038 | ARA12; subtilase [*Arabidopsis thaliana*] | Chaperones and protease |
| GLL010_F06_019 | Host specificity protein [*Methanococcoides burtonii* DSM 6242 | Non classified |
| GLL031_C04_014 | Sucrose-binding protein 2 [*Glycine max* | Carbohydrate |
| GLE094_C01_006 | Unknown [*Arabidopsis thaliana*] | Non classified |
| GLE021_H04_009 | PPF-1 protein [*Pisum sativum*] | Structure and development cycle |
| GLL054_A10_040 | Putative 1-deoxy-D-xylulose 5-phosphate reductoisomerase [*Zea mays*] | Energy |
| GLL094_D08_029 | Protein binding [*Arabidopsis thaliana*] | Non classified |
| GLE062_E02_004 | Putative 50S ribosomal protein L20 [*Oryza sativa* (japonica cultivar-group) | Translation |
| GLE016_B04_015 | RNA binding [*Arabidopsis thaliana*] | Transcription |
| GLE094_G01_002 | Protein binding / ubiquitin-protein ligase/ zinc ion binding [*Arabidopsis thaliana*] | Chaperones and protease |
| GLL057_D04_013 | ER lumen protein-retaining receptor-like [*Oryza sativa* (japonica cultivar-group)] | Signal transduction |
| GLE057_B04_015 | 60S acidic ribosomal protein P0 | Translation |
| GLE041_B08_031 | Structural constituent of ribosome [*Arabidopsis thaliana*] | Translation |
| GLE047_E04_012 | Unknown protein [*Arabidopsis thaliana*] | Non classified |
| GLE032_F06_019 | Unknown protein [*Arabidopsis thaliana*] | Non classified |
| GLE041_B08_031 | Structural constituent of ribosome [*Arabidopsis thaliana*] | Translation |
| GLE041_E10_036 | Putative Ruv DNA-helicase [*Cicer arietinum*] | Replication |

**Supplementary Table 3**: The length of the mature miRNAs in cluster bean

| **Group I**  (19 nucleotide long) | **Group II**  (20 nucleotide long) | **Group III**  (21nucleotide long) |
| --- | --- | --- |
| miR1132, miR1533,miR1535, miR2919, miR399, miR5565 | miR1044, miR1167, miR1527, miR156, miR168, miR2098, miR2105, miR2628, miR2866, miR3979, miR4413, miR5021, miR5265, miR5662 | miR1109, miR1313, miR1439, miR169, miR172, miR1852, miR1857, miR2082, miR2275, miR2606, miR2634, miR393, miR396, miR397, miR414, miR437, miR444, miR4995, miR5015, miR5338, miR537, miR5489, miR5542, miR5641, miR5658, miR773, miR779, miR781, miR837, miR838, miR863, miR865, miR867 |

**Supplementary Table 4: Calculated A/U** and C/G ratios in predicted miRNA families of cluster bean

| **Predicted miRNA Family** | **A/U** | **C/G** |
| --- | --- | --- |
| miR1044 | 0.565217 | 0.769231 |
| miR1109 | 0.722222 | 0.73913 |
| miR1132 | 0.833333 | 0.625 |
| miR1134 | 1.176471 | 1.111111 |
| miR1167 | 1.076923 | 0.727273 |
| miR1313 | 0.742857 | 0.941176 |
| miR1439 | 1.030043 | 1.36 |
| miR1527 | 1.636364 | 1.428571 |
| miR1533 | 1.206897 | 0.625 |
| miR1535 | 0.564103 | 1.04878 |
| miR156 | 1.263158 | 1.096774 |
| miR168 | 1.090909 | 0.923077 |
| miR169 | 1.0625 | 0.923077 |
| miR172 | 1.1875 | 1.333333 |
| miR1852 | 0.730769 | 0.636364 |
| miR1857 | 0.529412 | 0.461538 |
| miR2082 | 0.666667 | 0.421053 |
| miR2098 | 0.5 | 0.5 |
| miR2105 | 0.810811 | 0.727273 |
| miR2275 | 1.066667 | 1.125 |
| miR2606 | 0.769231 | 0.888889 |
| miR2628 | 0.954545 | 0.428571 |
| miR2634 | 0.625 | 0.8 |
| miR2866 | 0.44186 | 1 |
| miR2919 | 0.954545 | 0.5625 |
| miR393 | 0.647059 | 0.846154 |
| miR396 | 0.5 | 0.9 |
| miR397 | 0.444444 | 1.2 |
| miR3979 | 0.6875 | 1.25 |
| miR399 | 1.4 | 0.8 |
| miR414 | 1.619048 | 0.965517 |
| miR437 | 0.947368 | 0.625 |
| miR4413 | 0.677419 | 0.411765 |
| miR444 | 0.363636 | 0.7 |
| miR4995 | 0.928571 | 1 |
| miR5015 | 0.391304 | 0.8 |
| miR5021 | 1.090909 | 0.642857 |
| miR5079 | 0.714286 | 1.25 |
| miR5265 | 1.411765 | 0.818182 |
| miR5267 | 0.913043 | 0.904762 |
| miR5338 | 1.058824 | 0.703125 |
| miR537 | 0.666667 | 0.2 |
| miR5489 | 1.125 | 0.722222 |
| miR5542 | 0.75 | 0.366667 |
| miR5565 | 0.76087 | 0.606061 |
| miR5641 | 0.733333 | 0.571429 |
| miR5658 | 0.636364 | 0.142857 |
| miR5662 | 1.44 | 0.879121 |
| miR773 | 0.375 | 0.583333 |
| miR779 | 0.707317 | 0.545455 |
| miR781 | 0.428571 | 0.555556 |
| miR837 | 0.659091 | 1 |
| miR838 | 0.958333 | 0.926667 |
| miR863 | 0.866667 | 0.777778 |
| miR865 | 1.222222 | 2 |
| miR867 | 1.285714 | 1 |
| miR902 | 0.909091 | 0.727273 |

**Supplementary Table 5: GO IDs and their annotated functions in different categories**

| **GO ID** | **Biotic stress/**  **Function** | **GO ID** | **Abiotic stress/**  **function** | **GO ID** | **Defense related/function** | **GO ID** | **Carbohydrate related function** |
| --- | --- | --- | --- | --- | --- | --- | --- |
| GO:0009615 | response to virus | GO:0006950 | response to stress | GO:0009867 | jasmonic acid-mediated signalling pathway | GO:0004560 | alpha-L-fucosidase activity |
| GO:0009616 | virus-induced gene silencing | GO:0009408 | response to heat | GO:0009862 | systemic acquired resistance, salicylic acid mediated signaling pathway | GO:0004575 | sucrose alpha-glucosidase activity |
| GO:0009617 | response to bacterium | GO:0009414 | response to water deprivation | GO:0009870 | defense response signaling pathway, resistance gene-dependent | GO:0004615 | phosphomannomutase activity |
| GO:0009624 | response to nematode | GO:0009615 | response to virus | GO:0009626 | plant-type hypersensitive response | GO:0004634 | phosphopyruvate hydratase activity |
| GO:0009816 | defense response to bacterium, incompatible interaction | GO:0009631 | cold acclimation | GO:0009751 | response to salicylic acid stimulus | GO:0005975 | carbohydrate metabolic process |
| GO:0009817 | defense response to fungus, incompatible interaction | GO:0009651 | response to salt stress |  |  | GO:0019307 | mannose biosynthetic process |
| GO:0035195 | gene silencing by miRNA | GO:0006979 | response to oxidative stress |  |  |  |  |
| GO:0042742 | defense response to bacterium | GO:0009409 | response to cold |  |  |  |  |

**Supplementary Table 6**: Description of target genes involved in the regulatory network using GENEMANIA software

| Gene name | Description of gene and its function |
| --- | --- |
| STO | B-box zinc finger family protein; Acts as a negative regulator of seedling photomorphogenesis and light-regulated inhibition of hypocotyl elongation. |
| AT1G12310 | Calcium-binding EF-hand family protein; Potential calcium sensor |
| MIOX1 | Myo-inositol oxygenase 1; Involved in the biosynthesis of UDP-glucuronic acid (UDP-GlcA) |
| SCL1 | Encodes a scarecrow-like protein (SCL1). Member of GRAS gene family (593 aa) |
| RD21A | Granulin repeat cysteine protease family protein; Cysteine protease that plays a role in immunity, senescence, and biotic and abiotic stresses (Probable). |
| AGO1 | Stabilizer of iron transporter SufD / Polynucleotidyl transferase; Involved in RNA-mediated post-transcriptional gene silencing (PTGS). |
| PGSIP4 | Putative UDP-glucuronate-xylan alpha-glucuronosyltransferase 4; May be involved in the substitutions of the xylan backbone in stem glucuronoxylan (557 aa) |
| A/N-InvA | Alkaline/neutral invertase A, mitochondrial; Mitochondrial invertase that cleaves sucrose into glucose and fructose and is involved in the regulation of multiple tissue development and floral transition. May generate glucose as a substrate for mitochondria-associated hexokinase, contributing to mitochondrial reactive oxygen species homeostasis (616 aa) |
| AT1G60420 | DC1 domain-containing protein; Probable thiol-disulfide oxidoreductase required for pollen tube growth and pollen function in the pistil. |
| TIM13 | Mitochondrial import inner membrane translocase subunit TIM13; |
| PTR6 | Major facilitator superfamily protein; Its function is described as transporter activity |
| ADH1 | Alcohol dehydrogenase class-P; |
| LACS9 | Long chain acyl-CoA synthetase 9, chloroplastic; Activation of long-chain fatty acids for both synthesis of cellular lipids, and degradation via beta-oxidation. |
| SQN | Peptidyl-prolyl cis-trans isomerase / cyclophilin-40 (CYP40) / rotamase; PPIases accelerate the folding of proteins. |
| MIOX2 | Myo-inositol oxygenase 2; Involved in the biosynthesis of UDP-glucuronic acid (UDP-GlcA), providing nucleotide sugars for cell-wall polymers. |
| AGO4 | Argonaute family protein; |
| CKS1 | Catalytic subunit of cyclin dependent kinase 1 |
| AR17 | Probable E3 ubiquitin-protein ligase ARI7; |
| CRK1 | CDPK-related kinase 1; May play a role in signal transduction pathways that involve calcium as a second messenger (By similarity). Serine/threonine kinase that phosphorylates histone H3.) |
| AT2G41410 | Calcium-binding EF-hand family protein; Potential calcium sensor. |
| PMM | Phosphomannomutase; Involved in ascorbic acid biosynthesis |
| ASD1 | Alpha-L-arabinofuranosidase 1; May be involved in the coordinated dissolution of the cell wall matrix during abscission and in the secondary cell wall formation in xylem vessels. |
| EBP | Ethylene-responsive transcription factor RAP2-3; |
| ADF2 | Actin-depolymerizing factor 2. Required for normal cell growth, plant development, cell organ expansion and flowering. Essential for root-knot nematode infection. |
| AT3G49810 | U-box domain-containing protein 30; Functions as an E3 ubiquitin ligase. |
| EXPA17 | Putative expansin-A17; Causes loosening and extension of plant cell walls by disrupting non-covalent bonding between cellulose microfibrils and matrix glucans. |
| CUL1 | Cullin-1; Involved in ubiquitination and subsequent proteasomal degradation of target proteins. |
| MSRB3 | Peptide methionine sulfoxide reductase B3; Catalyzes the reduction of methionine sulfoxide (MetSO) to methionine in proteins. Plays a protective role against oxidative stress by restoring activity to proteins that have been inactivated by methionine oxidation. Involved in cold tolerance. |
| CLA1 | 1-deoxy-D-xylulose-5-phosphate synthase, chloroplastic; Catalyzes the acyloin condensation reaction between C atoms 2 and 3 of pyruvate and glyceraldehyde 3-phosphate to yield 1-deoxy-D-xylulose-5-phosphate (DXP). Is a limiting enzyme for plastidic isoprenoid biosynthesis and essential for chloroplast development |
| AG | K-box region and MADS-box transcription factor family protein; Probable transcription factor involved in the control of organ identity during the early development of flowers. Is required for normal development of stamens and carpels in the wild-type flower. Plays a role in maintaining the determinacy of the floral meristem. |
| NRPB2 | DNA-directed RNA polymerase family protein |
| Tic32-IVa | NAD(P)-binding Rossmann-fold superfamily protein; Involved in protein precursor import into chloroplasts. |
| LRE | GPI-anchored protein LORELEI; Female gametophyte-specific component of the signaling pathway required for fertilization. Required for the reception of the pollen tube by the female gametophyte. |
| PCK1 | Encodes a putative phosphoenolpyruvate carboxykinase (ATP-dependent) (671 aa) |
| SNX1 | Sorting nexin 1; Plays a role in vesicular protein sorting. |
| ASD2 | Alpha-L-arabinofuranosidase 2; May be involved in the coordinated dissolution of the cell wall matrix during abscission and in the secondary cell wall formation in xylem vessels; |
| CKS3 | Cytokinin dehydrogenase 3; Catalyzes the oxidation of cytokinins |
| YKT61 | SNARE-like superfamily protein; Member of YKT6 Gene Family |

**Supplementary Table 7: List of different transcription factor identified from c**luster bean ESTs

| **Transcription**  **factor symbol** | **EST ID** | **Arabidopsis**  **thaliana ID** | **E-value** | **Transcription factor description** |
| --- | --- | --- | --- | --- |
| AP2 | EG978135.1 | AT5G10510.1 | 4.00E-35 | AINTEGUMENTA-like 6 |
| B3 | EG988153.1 | AT3G53310.1 | 1.00E-21 | B3 family protein |
| B3 | EG988477.1 | AT3G53310.1 | 2.00E-13 | B3 family protein |
| B3 | EG989650.1 | AT3G53310.1 | 3.00E-19 | B3 family protein |
| BBR-BPC | EG988152.1 | AT5G42520.1 | 5.00E-35 | basic pentacysteine 6 |
| BBR-BPC | EG989811.1 | AT2G21240.1 | 4.00E-37 | basic pentacysteine 4 |
| bHLH | EG976672.1 | AT4G36930.1 | 8.00E-43 | bHLH family protein |
| bHLH | EG979132.1 | AT4G02590.3 | 1.00E-81 | bHLH family protein |
| bHLH | EG982170.1 | AT4G36930.1 | 1.00E-37 | bHLH family protein |
| bHLH | EG985083.1 | AT5G62610.1 | 2.00E-48 | bHLH family protein |
| bZIP | EG984194.1 | AT5G60830.1 | 2.00E-31 | basic leucine-zipper 70 |
| bZIP | EG984645.1 | AT1G75390.2 | 2.00E-20 | basic leucine-zipper 44 |
| bZIP | EG985556.1 | AT5G60830.1 | 3.00E-31 | basic leucine-zipper 70 |
| bZIP | EG985629.1 | AT5G60830.1 | 3.00E-31 | basic leucine-zipper 70 |
| bZIP | EG986305.1 | AT5G60830.1 | 1.00E-29 | basic leucine-zipper 70 |
| bZIP | EG986350.1 | AT5G60830.1 | 3.00E-31 | basic leucine-zipper 70 |
| bZIP | EG986645.1 | AT3G12250.1 | 3.00E-27 | TGACG motif-binding factor 6 |
| bZIP | EG986785.1 | AT5G60830.1 | 5.00E-29 | basic leucine-zipper 70 |
| bZIP | EG987094.1 | AT5G60830.1 | 2.00E-31 | basic leucine-zipper 70 |
| bZIP | EG987745.1 | AT3G12250.1 | 5.00E-32 | TGACG motif-binding factor 6 |
| bZIP | EG987841.1 | AT5G60830.1 | 3.00E-31 | basic leucine-zipper 70 |
| bZIP | EG989634.1 | AT3G62420.1 | 3.00E-30 | basic region/leucine zipper motif 53 |
| bZIP | EG990212.1 | AT2G40620.1 | 7.00E-54 | bZIP family protein |
| C2H2 | EG986248.1 | AT1G72050.2 | 3.00E-58 | transcription factor IIIA |
| C3H | EG982280.1 | AT2G19810.1 | 2.00E-42 | C3H family protein |
| C3H | EG983965.1 | AT4G29190.1 | 2.00E-91 | C3H family protein |
| C3H | EG984169.1 | AT2G19810.1 | 2.00E-80 | C3H family protein |
| C3H | EG984216.1 | AT3G12130.1 | 1.00E-27 | C3H family protein |
| C3H | EG986530.1 | AT2G20280.1 | 1.00E-118 | C3H family protein |
| C3H | EG987165.1 | AT3G48440.1 | 4.00E-55 | C3H family protein |
| C3H | EG989120.1 | AT3G51120.1 | 2.00E-09 | DNA binding;zinc ion binding;nucleic  acid binding;nucleic acid binding |
| C3H | EG989906.1 | AT3G12680.1 | 3.00E-69 | floral homeotic protein (HUA1) |
| C3H | EG990338.1 | AT2G47850.2 | 2.00E-36 | C3H family protein |
| DBB | EG987192.1 | AT1G06040.1 | 5.00E-80 | DBB family protein |
| Dof | EG983224.1 | AT3G47500.1 | 3.00E-56 | cycling DOF factor 3 |
| Dof | EG987238.1 | AT5G60200.1 | 2.00E-37 | TARGET OF MONOPTEROS 6 |
| Dof | EG988750.1 | AT3G61850.4 | 2.00E-43 | Dof family protein |
| ERF | EG979339.1 | AT1G53910.3 | 5.00E-45 | related to AP2 12 |
| ERF | EG987232.1 | AT2G40350.1 | 2.00E-51 | ERF family protein |
| ERF | EG989710.1 | AT5G25390.2 | 2.00E-66 | ERF family protein |
| FAR1 | EG987116.1 | AT3G06250.1 | 8.00E-11 | FAR1-related sequence 7 |
| G2-like | EG989991.1 | AT3G24120.1 | 9.00E-46 | G2-like family protein |
| G2-like | EG990581.1 | AT3G46640.2 | 6.00E-56 | G2-like family protein |
| GATA | EG984775.1 | AT4G17570.1 | 6.00E-41 | GATA transcription factor 26 |
| GRAS | EG980044.1 | AT1G21450.1 | 1.00E-124 | SCARECROW-like 1 |
| GRAS | EG983395.1 | AT1G66350.1 | 0.001 | RGA-like 1 |
| GRAS | EG986533.1 | AT1G07530.1 | 6.00E-27 | SCARECROW-like 14 |
| GRAS | EG987172.1 | AT1G66350.1 | 7.00E-26 | RGA-like 1 |
| HB-other | EG984544.1 | AT2G22430.1 | 1.00E-36 | homeobox protein 6 |
| HB-other | EG985729.1 | AT3G18380.1 | 2.00E-50 | sequence-specific DNA binding transcription  factors;sequence-specific DNA binding |
| HB-other | EG986732.1 | AT4G00730.2 | 1.00E-71 | HD-ZIP family protein |
| HB-other | EG989013.1 | AT4G37790.1 | 2.00E-34 | HD-ZIP family protein |
| HB-other | EG991072.1 | AT4G37790.1 | 7.00E-34 | HD-ZIP family protein |
| HSF | EG984522.1 | AT5G62020.1 | 9.00E-64 | heat shock transcription factor B2A |
| LBD | EG983845.1 | AT4G37540.1 | 3.00E-50 | LOB domain-containing protein 39 |
| LBD | EG984806.1 | AT4G37540.1 | 7.00E-43 | LOB domain-containing protein 39 |
| MIKC_MADS | EG978090.1 | AT3G58780.1 | 1.00E-104 | MIKC_MADS family protein |
| MIKC_MADS | EG978874.1 | AT3G58780.1 | 1.00E-112 | MIKC_MADS family protein |
| MIKC_MADS | EG979876.1 | AT3G58780.1 | 1.00E-100 | MIKC_MADS family protein |
| MIKC_MADS | EG981212.1 | AT3G58780.1 | 1.00E-101 | MIKC_MADS family protein |
| MIKC_MADS | EG983575.1 | AT2G42830.1 | 1.00E-107 | MIKC_MADS family protein |
| MIKC_MADS | EG984171.1 | AT5G15800.1 | 3.00E-76 | MIKC_MADS family protein |
| MIKC_MADS | EG985012.1 | AT3G58780.1 | 1.00E-113 | MIKC_MADS family protein |
| MIKC_MADS | EG985114.1 | AT3G58780.1 | 1.00E-111 | MIKC_MADS family protein |
| MIKC_MADS | EG985558.1 | AT5G15800.1 | 6.00E-92 | MIKC_MADS family protein |
| MIKC_MADS | EG985619.1 | AT3G58780.1 | 1.00E-112 | MIKC_MADS family protein |
| MIKC_MADS | EG986489.1 | AT4G18960.1 | 1.00E-115 | MIKC_MADS family protein |
| MIKC_MADS | EG986495.1 | AT3G58780.1 | 1.00E-111 | MIKC_MADS family protein |
| MIKC_MADS | EG986583.1 | AT3G58780.1 | 1.00E-109 | MIKC_MADS family protein |
| MIKC_MADS | EG987157.1 | AT4G18960.1 | 1.00E-107 | MIKC_MADS family protein |
| MIKC_MADS | EG988262.1 | AT2G42830.1 | 1.00E-111 | MIKC_MADS family protein |
| MIKC_MADS | EG988715.1 | AT3G58780.1 | 1.00E-109 | MIKC_MADS family protein |
| MIKC_MADS | EG988930.1 | AT3G58780.1 | 1.00E-114 | MIKC_MADS family protein |
| MIKC_MADS | EG989317.1 | AT3G54340.1 | 1.00E-63 | MIKC_MADS family protein |
| MIKC_MADS | EG989413.1 | AT1G24260.1 | 1.00E-108 | MIKC_MADS family protein |
| MIKC_MADS | EG990135.1 | AT3G58780.1 | 1.00E-110 | MIKC_MADS family protein |
| MIKC_MADS | EG990361.1 | AT4G18960.1 | 1.00E-108 | MIKC_MADS family protein |
| MIKC_MADS | EG990579.1 | AT2G42830.1 | 1.00E-110 | MIKC_MADS family protein |
| MIKC_MADS | EG990688.1 | AT1G24260.2 | 1.00E-117 | MIKC_MADS family protein |
| MIKC_MADS | EG991168.1 | AT3G54340.1 | 7.00E-64 | MIKC_MADS family protein |
| MIKC_MADS | EG991235.1 | AT5G13790.1 | 4.00E-54 | AGAMOUS-like 15 |
| M-type_MADS | EG988204.1 | AT3G02310.1 | 1.00E-65 | MIKC_MADS family protein |
| MYB | EG984266.1 | AT5G15310.2 | 1.00E-63 | myb domain protein 16 |
| MYB | EG987098.1 | AT5G35550.1 | 7.00E-65 | MYB family protein |
| MYB | EG987114.1 | AT2G37630.1 | 8.00E-88 | MYB family protein |
| MYB | EG988716.1 | AT3G11450.1 | 2.00E-77 | DnaJ domain ;Myb-like DNA-binding domain |
| MYB_related | EG985219.1 | AT1G01060.3 | 1.00E-63 | MYB_related family protein |
| NAC | EG975444.1 | AT1G69490.1 | 3.00E-34 | NAC-like, activated by AP3/PI |
| NAC | EG980141.1 | AT1G69490.1 | 3.00E-34 | NAC-like, activated by AP3/PI |
| NAC | EG984599.1 | AT3G10480.1 | 8.00E-93 | NAC domain containing protein 50 |
| NAC | EG987989.1 | AT5G04410.1 | 6.00E-92 | NAC domain containing protein 2 |
| NF-YA | EG986414.1 | AT1G30500.2 | 1.00E-61 | nuclear factor Y, subunit A7 |
| NF-YB | EG975780.1 | AT2G27470.1 | 5.00E-15 | nuclear factor Y, subunit B11 |
| NF-YB | EG977168.1 | AT5G47670.2 | 7.00E-26 | nuclear factor Y, subunit B6 |
| NF-YB | EG981498.1 | AT4G14540.1 | 4.00E-64 | nuclear factor Y, subunit B3 |
| NF-YB | EG987836.1 | AT5G23090.2 | 5.00E-94 | nuclear factor Y, subunit B13 |
| NF-YC | EG989948.1 | AT1G56170.1 | 4.00E-52 | nuclear factor Y, subunit C2 |
| Nin-like | EG983635.1 | AT4G24020.1 | 2.00E-28 | NIN like protein 7 |
| S1Fa-like | EG978767.1 | AT2G37120.1 | 9.00E-15 | S1FA-like DNA-binding protein |
| S1Fa-like | EG987760.1 | AT2G37120.1 | 9.00E-15 | S1FA-like DNA-binding protein |
| SBP | EG982824.1 | AT1G53160.1 | 1.00E-31 | squamosa promoter binding protein-like 4 |
| TALE | EG978095.1 | AT5G11060.1 | 7.00E-61 | KNOTTED1-like homeobox gene 4 |
| TCP | EG980264.1 | AT5G23280.1 | 1.00E-20 | TCP family protein |
| TCP | EG986580.1 | AT3G15030.2 | 4.00E-60 | TCP family protein |
| TCP | EG989929.1 | AT5G08330.1 | 2.00E-26 | TCP family protein |
| TCP | EG989942.1 | AT5G08330.1 | 2.00E-26 | TCP family protein |
| Trihelix | EG983255.1 | AT2G44730.1 | 1.00E-54 | Trihelix family protein |
| WRKY | EG984018.1 | AT2G04880.1 | 3.00E-27 | zinc-dependent activator protein-1 |
| WRKY | EG989703.1 | AT2G23320.1 | 1.00E-68 | WRKY DNA-binding protein 15 |
| YABBY | EG977552.1 | AT1G08465.1 | 6.00E-78 | YABBY family protein |
| ZF-HD | EG990895.1 | AT3G28917.1 | 8.00E-38 | mini zinc finger 2 |

**Supplementary Figures**


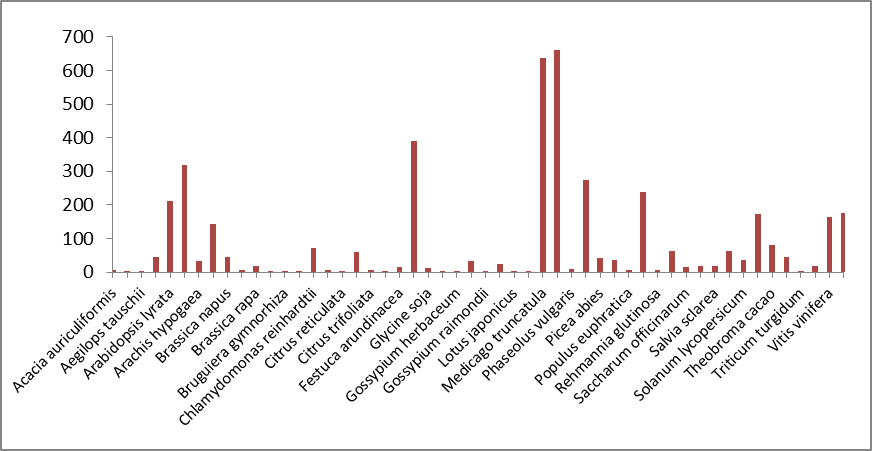


**Supplementary Figure 1: Graph showing mature miRNA from various plant species in miRBase**


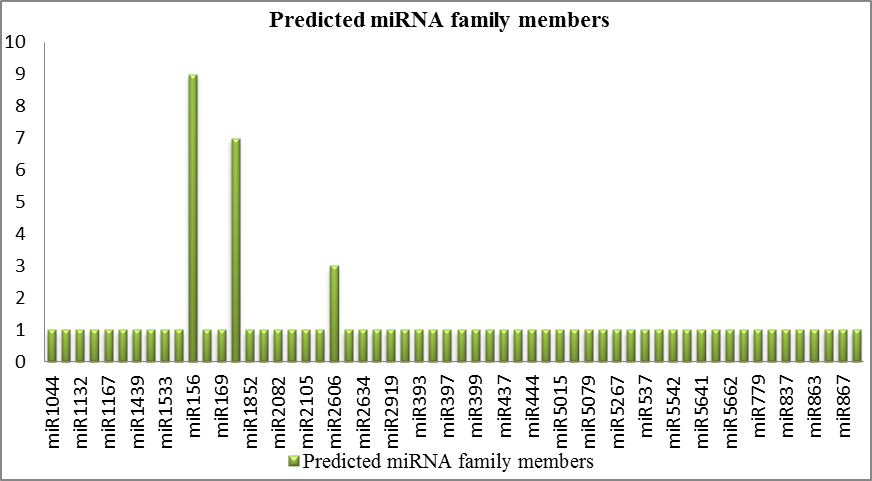


**Supplementary Figure 2: Number of members in Predicted miRNA Families of cluster bean**


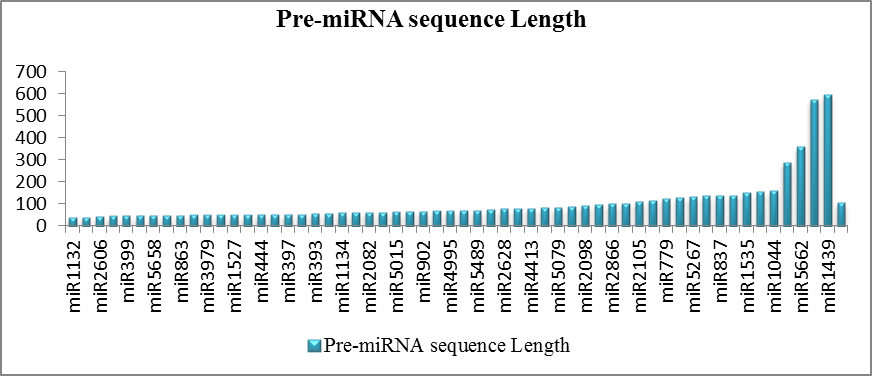


**Supplementary Figure 3: Pre-miRNA sequence length of predicted miRNA family members in cluster bean**


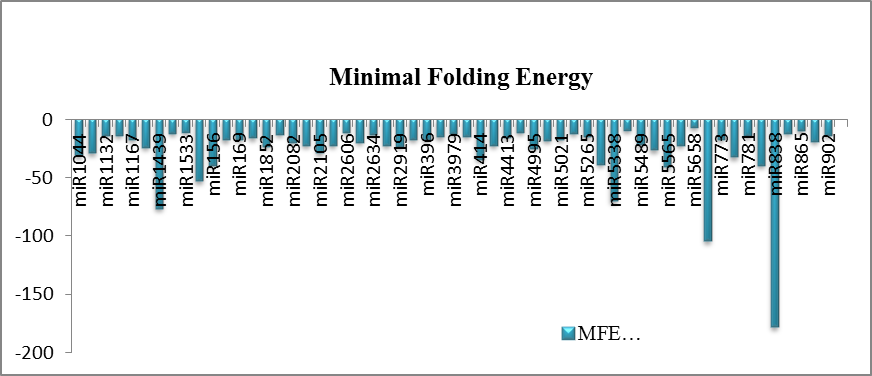


**Supplementary Figure 4: Minimal folding free energy range for predicted miRNA families in cluster bean**

**
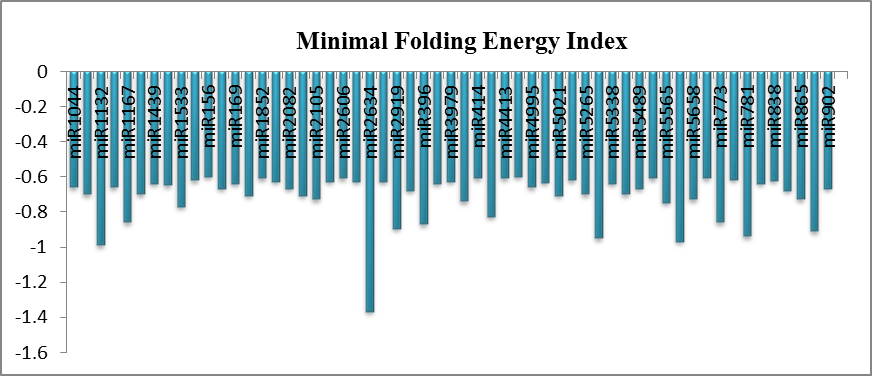
**

**Supplementary Figure 5: Minimal folding free energy index for predicted miRNA families in cluster bean**


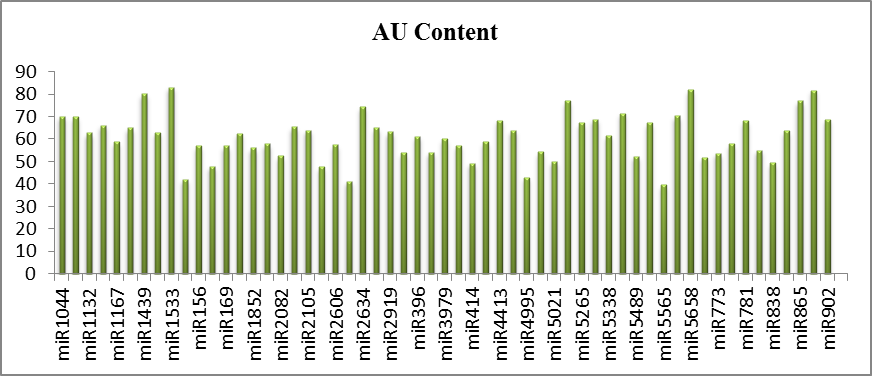


**Supplementary Figure 6: AU content of predicted miRNA families in cluster bean**


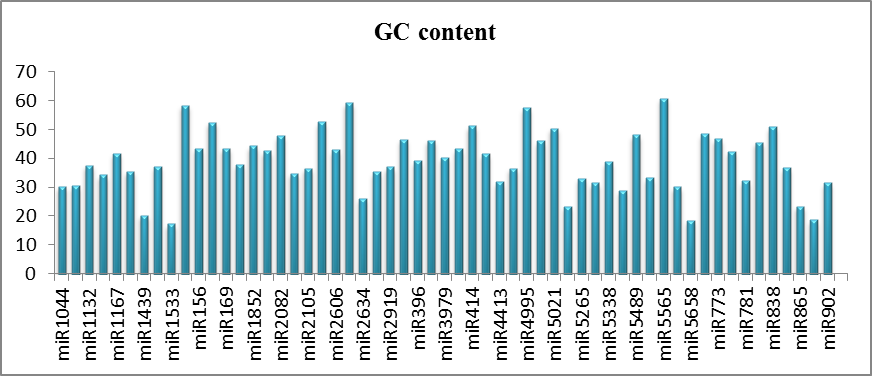


**Supplementary Figure 7: GC content of predicted miRNA families in cluster bean**


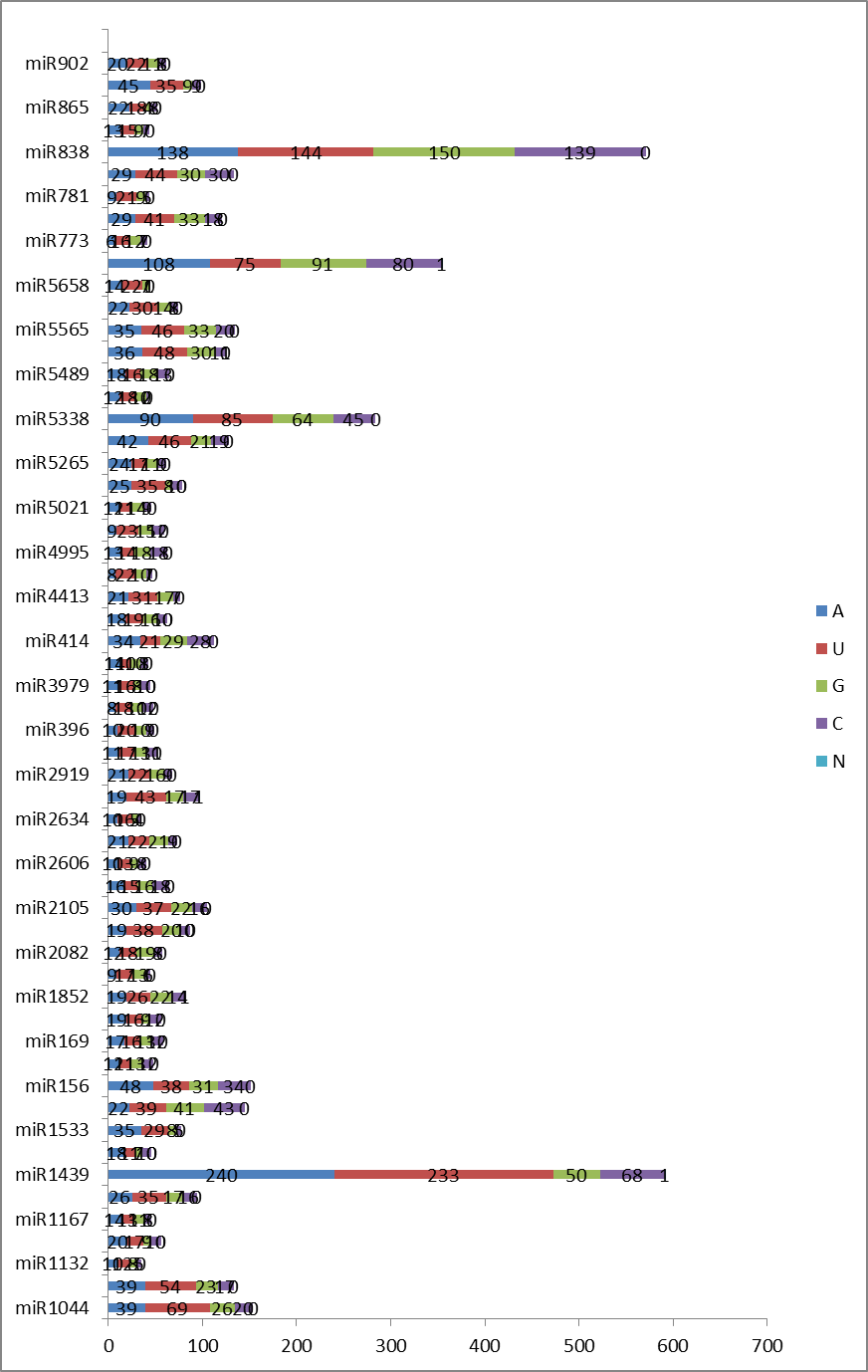
**Supplementary Figure 8: Nucleotide content for different predicted miRNA families in cluster bean**
